# The binding structure of event elements in episodic memory and the role of animacy

**DOI:** 10.1177/17470218221096148

**Published:** 2022-06-09

**Authors:** Marcel R Schreiner, Thorsten Meiser, Arndt Bröder

**Affiliations:** Department of Psychology, School of Social Sciences, University of Mannheim, Mannheim, Germany

**Keywords:** Episodic memory, binding, animacy, statistical modelling

## Abstract

Experienced events consist of several elements which need to be bound together in memory to represent the event in a coherent manner. Given such bindings, the retrieval of one event element should be related to the successful retrieval of another element of the same event, thus leading to a stochastic dependency of the retrieval of event elements. The way in which bindings are structured is not yet clearly established and only few moderators of the binding of event elements have been identified. We present results from three experiments aiming to distinguish between an integrated binding structure, in which event elements are bound into a unitary representation, and a hierarchical binding structure, in which event elements are preferentially bound to specific types of elements. Experiments 2 and 3 were additionally designed to identify animacy, an entity’s property of being alive, as a potential moderator of the binding of event elements. We also offer a new approach for modelling dependencies of the retrieval of event elements which mitigates some limitations of previous approaches. Consistent with previous literature, we found dependencies of the retrieval of event elements if all of an event’s constituent associations were shown. We found mixed evidence for integrated or hierarchical binding structures but found dependency of the retrieval of event elements to be sensitive to the presence of animacy in an event. The results suggest that binding structures may vary depending on moderators such as animacy or event structure awareness. Theoretical implications and directions for future research are discussed.

Episodic memory stores information about experienced events (Tulving, 1972, 1983) which consist of multiple elements, such as persons, objects, locations, actions, and sensations. Despite different event elements being represented in different neocortical regions (Alvarez & Squire, 1994; Horner et al., 2015), they need to be bound together to enable the retrieval of the event in a coherent manner. The hippocampus is considered to be the structure responsible for accomplishing this task (Backus et al., 2016; Cohen & Eichenbaum, 1993; Davachi et al., 2003; Eichenbaum et al., 2007; Squire & Zola-Morgan, 1991). Binding should be associated with an increased likelihood of retrieving subsequent event elements if a preceding element was successfully retrieved. This leads to a stochastic dependency of the retrieval of event elements (e.g., Arnold et al., 2019; Boywitt & Meiser, 2012a, 2012b; Bröder, 2009; Horner & Burgess, 2013, 2014; Horner et al., 2015; Meiser & Bröder, 2002; Ngo et al., 2019; Starns & Hicks, 2005, 2008) whereas it is not precluded that dependency is affected by retrieval-based processes in addition to binding processes occurring during encoding, such as suggested by Kumaran and McClelland (2012). However, there exist diverging views regarding the representational structure in which different event elements are bound together.

One purpose of the current research is to distinguish between an integrated binding structure, in which event elements are bound into a unitary representation, and a hierarchical binding structure, in which event elements are preferentially bound to particular elements. This relates to the fundamental principles driving information storage and retrieval in episodic memory. Some authors suggest that the hippocampus acts as a convergence zone, binding event elements into a single engram which can then be retrieved by partial activation of event elements via pattern completion (Damasio, 1989; Marr, 1971; Moll & Miikkulainen, 1997). This is consistent with Tulving’s idea of event engrams as discrete bound event representations, containing information about different event elements (Tulving, 1983). A related view is integrative encoding, which suggests that the hippocampus integrates newly encountered associations into existing, overlapping, ones, ultimately leading to an integrated representation containing all event elements (Shohamy & Wagner, 2008; Zeithamova et al., 2012). We term such representations or engrams an integrated binding structure. Thus, in an integrated binding structure, elements of a given event and associations among these elements are represented in a single superordinate memory structure and can consequently be accessed in an all-or-none manner. However, results supporting integrative encoding may also be explained by pairwise, non-overlapping, representations of individual experiences (Kumaran & McClelland, 2012; McClelland et al., 1995). In addition, other views, such as ensemble encoding (Cai et al., 2016), relational memory theory (Cohen & Eichenbaum, 1993; Eichenbaum, 1999; see also Eichenbaum & Cohen, 1988, 2001), and the theory of event coding (TEC, Hommel et al., 2001), more strongly emphasise pairwise representations. Ensemble encoding posits that associations are stored as overlapping ensembles while remaining distinct rather than forming a unitary representation (Cai et al., 2016). Relational memory theory suggests that the hippocampus flexibly links event elements such that they can be recombined depending on task demands. In the TEC, codes of stimuli (feature codes) are activated upon perception and are then bound into so-called event files (Hommel, 1998, 2009). Event files do not consist of a unitary representation but rather of multiple local interconnections as a result of selective binding (Hommel, 1998, 2004). In addition, the degree to which feature codes contribute to the event file may vary (Hommel et al., 2001) and not all possible pairwise bindings are necessarily formed (Moeller et al., 2019). The TEC thus allows for an asymmetry of bindings in event files. This may explain findings of asymmetries in the retrieval of event elements, such that some types of elements serve as more effective retrieval cues or are retrieved more likely (e.g., Hayes et al., 2004; Nairne et al., 2017; Trinkler et al., 2006). Binding asymmetries are also possible in the recently proposed Span–Cospan model of episodic memory (Healy & Caudell, 2019). When events are presented as sequences of event segments, the model assumes that event elements form higher order representations of event segments which are represented by specific cells. The representations may consequently form further higher level representations up to a representation of the entire event while holistic access to individual event segments is maintained. Representations and connections can vary in strength. Thus, asymmetries are possible if the connection strength of cells responsible for representations at different levels varies such that certain combinations of event elements lead to stronger higher level representations. From these views, it follows that bindings may be hierarchically organised such that event elements are preferentially bound to one type of element. Thus, a hierarchical binding structure does not posit that event elements are represented in a unitary manner but rather that they are organised in a system of pairwise bindings in which some bindings may be systematically prioritised over others, allowing for asymmetries in binding strength.

The distinction between an integrated and a hierarchical binding structure is related to the discussion of the binding variability and the mutual cuing hypothesis in the source memory literature, which refers to memory for the conditions under which a memory has been acquired (Johnson et al., 1993). The binding variability hypothesis suggests that source features are primarily bound to the item rather than to each other (Starns & Hicks, 2005; see also the model of headed records, J. Morton et al., 1985), pointing to an item–feature hierarchy. The mutual cuing hypothesis suggests additional direct binding of features (Meiser & Bröder, 2002), which makes it more similar to an integrated binding structure. However, the mutual cuing hypothesis does not necessarily predict that item and features are bound into a unitary representation. There is an ongoing debate regarding the two accounts, with some results supporting mutual cuing (Boywitt & Meiser, 2012a, 2012b; Meiser & Bröder, 2002; see also Balaban et al., 2019) and others supporting binding variability (Hicks & Starns, 2016; Starns & Hicks, 2005, 2008; Vogt & Bröder, 2007). There is some evidence against an integrated binding structure in item-based representations as investigated in the source memory literature (Brady et al., 2013; Utochkin & Brady, 2020). Note, however, that item-based representations may differ from the more complex event-based representations that are the focus of the current research (Andermane et al., 2021; Brady et al., 2013; Joensen et al., 2020; Utochkin & Brady, 2020). Event-based representations consist of several elements, which can be considered to be item-based representations. Thus, item-based representations are nested within event-based representations (see Andermane et al., 2021). Item-based representations can also contain more specific information than event-based representations (Hunt & Einstein, 1981). Furthermore, event-based representations are potentially dynamic, include a spatiotemporal context, and allow for the construction of scenes, which is not the case for item-based representations (Andermane et al., 2021; Robin, 2018; Rubin & Umanath, 2015).

Direct behavioural evidence for integrated or hierarchical binding structures is scarce. Horner and Burgess (2013) found a dependency of the retrieval of event elements by having participants learn a series of events consisting of several elements (person, object, and location). For example, participants may be presented *David Cameron–bicycle–swimming pool*. Horner and Burgess (2014) and Horner et al. (2015) built on this procedure and introduced the separated encoding paradigm in which each pairwise association is presented separately during encoding. For example, given the previous example event with the elements *David Cameron*, *bicycle*, and *swimming pool*, participants may be presented the pairs *David Cameron–bicycle*, *bicycle–swimming pool*, and *swimming pool–David Cameron* across different learning trials (see also Figure 1a). Note that in this paradigm, different learning trials referring to the same event are not presented in sequence but are interleaved with learning trials referring to other events. While this may deviate to some extent from how events are “naturally” experienced, it allows to manipulate the associative structure of an event presentation (see Horner et al., 2015). Dependency in a separated encoding condition was not reduced compared with simultaneous encoding (but see James et al., 2020, for boundary conditions). However, this was only the case when all events were presented in a closed-loop (CL) structure in which all pairwise associations are shown (i.e., all possible pairings of event elements), but not in an open-loop structure in which the presentation of one pairwise association is excluded such that, for example, *David Cameron–bicycle* is not presented (Horner & Burgess, 2014; Horner et al., 2015; see also Joensen et al., 2020). The authors concluded that binding depends on the coherence of the encoding episode. These results seem to be in favour of an integrated binding structure. However, the authors did not systematically vary the excluded association within the open-loop condition. Thus, the specific association being excluded in an event could vary within the open-loop condition. We argue that, for testing an integrated against a hierarchical binding structure, it is necessary to systematically vary the excluded association across different experimental conditions (see also Cabeza, 2006). If this is not done, associations that may be critical for binding are excluded for some events but not for others within the same condition. In addition, if associations are not excluded systematically, it may be the last presented association that yields coherence, as found by Horner et al. (2015). However, this may be different if associations are excluded systematically. Thus, in the current research, we focus on associations between event elements irrespective of presentation order. We used several open-loop conditions in each of which only one type of association (e.g., object–location) was excluded from presentation (see also Figure 1c and d) instead of a single open-loop condition in which the type of excluded association could vary. In addition, Horner and Burgess (2014) and Horner et al. (2015) used an approach for modelling stochastic dependencies of the retrieval of event elements introduced by Horner and Burgess (2013). This approach is based on contingency tables for the retrieval of event elements in different test pairs (i.e., pairs of test trials in a memory test), which are aggregated across events.

**Figure 1.**
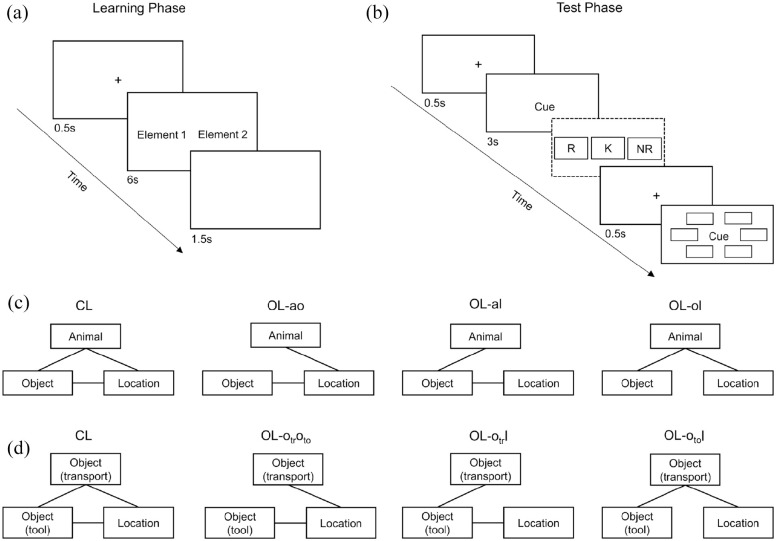
Experimental design and procedure. (a) Schematic depiction of a learning trial. (b) Schematic depiction of a test trial; recollection judgements (dashed rectangle) were only assessed in Experiment 1. (c) Associative structure of the experimental conditions in Experiment 1 and in the animacy condition of Experiments 2 and 3. (d) Associative structure of the non-animacy condition in Experiments 2 and 3. *Note.* R = remember; K = know; NR = no recognition; CL = closed loop; OL-ao = open loop with association animal–object excluded; OL-al = open loop with association animal–location excluded; OL-ol = open loop with association object–location excluded; OL- $o_{tr}o_{to}$ = open loop with association means of transportation–tool excluded; OL- $o_{tr}$ l = open loop with association means of transportation–location excluded; OL- $o_{to}$ l = open loop with association tool–location excluded; transport = means of transportation.

We propose a new approach for modelling the stochastic dependency of the retrieval of event elements based on item response theory (IRT; Lord, 1980; Lord & Novick, 1968) that takes individual item^1^ responses as input. The approach exploits the assumption of local independence (LI) inherent in many IRT models. LI requires item responses to be independent given a general latent person trait such as memory performance (de Ayala, 2009; Lazarsfeld & Henry, 1968). If binding of event elements occurs, this would result in event-specific effects which influence item responses in addition to the general latent person trait. This would violate the LI assumption and manifest in nonzero residual correlations for item pairs belonging to the same event. The estimated item residual correlations are used for computing the dependency measure, which contrasts the item residual correlations within events with the item residual correlations between events. The approach provides several advantages over previous approaches such as the one by Horner and Burgess (2013) or Yule’s *Q* (Yule, 1912; see also Hayman & Tulving, 1989; Horner & Burgess, 2014). It does not require the aggregation of responses into contingency tables and does not require the pre-specification of fixed test pairs, as is the case for previous approaches. In addition, our approach yields higher statistical power for detecting dependencies and differences in dependency between conditions than do previous approaches while providing good maintenance of Type I error rates (Schreiner & Meiser, 2022). Because previous approaches are based on aggregated contingency tables, they are prone to Simpson’s paradox (Hintzman, 1972, 1980; Simpson, 1951), stating that collapsing 2 × 2 contingency tables into summary ones may lead to relationships of the two outcome variables in the summary tables diverge from the ones in the original tables. This is not the case for our approach because it is not contingency-based. In addition, our approach can account for varying item difficulties and allows to account for guessing. It can, in principle, also be applied to a greater variety of test formats such as free recall and is not limited to cued recall or cued recognition.

Based on the results of our first experiment, we additionally aimed to identify animacy as a potential moderator of the binding of event elements. Such moderators have largely been absent in the literature so far. To our knowledge, the results by James et al. (2020), which hint at the modality of stimulus presentation (written vs. pictorial) and the dimensionality of presentation modality (unimodal vs. multimodal) to be potential moderators of the binding of event elements in the context of the separated encoding paradigm (Horner & Burgess, 2014; Horner et al., 2015), are the only ones referring to this topic.

In the current research, we aim to determine whether event elements are bound into an integrated or a hierarchical structure and investigate animacy as a potential moderator in the binding of event elements. Building on the work by Horner and Burgess (2014) and Horner et al. (2015), we aim to overcome limitations of earlier studies by systematically varying the excluded associations and offering a novel approach for modelling the stochastic dependency of the retrieval of event elements which mitigates some limitations of previous approaches. To this end, we conducted three experiments. The results of Experiment 1 are in favour of a hierarchical binding structure in which event elements are preferentially bound to an animate element. Experiments 2 and 3 were designed to both replicate and extend the findings from Experiment 1 by additionally investigating whether animacy influences the binding of event elements. While the results in favour of a hierarchical binding structure did not replicate across experiments, the experiments yielded evidence that animacy influences the binding of event elements.

## Experiment 1

In Experiment 1, we tested an integrated against a hierarchical binding account. We expected to replicate findings of a stochastic dependency of the retrieval of event elements (Hypothesis 1). In the source memory literature, a stochastic dependency of the retrieval of event elements has only been found for remember responses but not for know responses (Boywitt & Meiser, 2012a, 2012b; Meiser & Bröder, 2002; Meiser et al., 2008; Starns & Hicks, 2005). Remember and know responses are subjective ratings of memory quality, intended to tap into feelings of conscious recollection and experienced familiarity, respectively. While both recollection and familiarity enable recognition (Gardiner, 1988; Tulving, 1985), they may be different forms of memory with different functional characteristics (see Yonelinas, 2002, for a review). Similarly, we expected to only find a dependency of the retrieval of event elements in the case of recollection for event-based representations (Hypothesis 2). Previous findings suggest that dependency of the retrieval of event elements is eliminated if the encoding episode is not coherent (open-loop structure; Horner & Burgess, 2014; Horner et al., 2015). We suspected that effects may be masked because excluded associations in the open-loop condition were not systematically varied and due to limitations of the modelling approach used. While we expected that dependency is reduced in non-coherent encoding episodes, we did not expect that dependency is completely eliminated in such situations (Hypothesis 3). Finally, integrated and hierarchical binding structures make different predictions regarding dependency in non-coherent encoding episodes (open-loop structures), in which specific associations are excluded during study. An integrated binding structure suggests that the dependency does not vary as a function of the association being excluded. This is because an integrated binding structure consists of only a single unitary representation of the event that can be accessed in an all-or-none manner. Thus, the association that was not presented should readily be retrieved with the other associations from this unitary representation as if all associations were equally strong or retrieval should fail for all associations.^2^ On the contrary, a hierarchical binding structure does not posit a unitary representation and it suggests an asymmetry in the binding strength of event elements, leading to some associations being more critical for dependency than others. Thus, excluding an association should affect more critical associations in some cases, so that stochastic dependency is diminished, and less critical associations in others, so that stochastic dependency is preserved or diminished to a smaller extent. Consequently, a hierarchical binding structure suggests that dependency varies as a function of the excluded association (Hypothesis 4). The experiment was preregistered at https://osf.io/ncpvq.

### Method

#### Design

Each event consisted of the three constituent elements: animal, object, and location. There were four experimental within-subjects conditions (loop conditions). In the CL condition, all possible pairwise associations were presented (animal–object, animal–location, and object–location). In each of the three open-loop conditions, one pairwise association was consistently excluded from presentation (see also the paired-associate learning paradigm; e.g., Preston et al., 2004). Consequently, there was one condition in which animal–object was excluded (OL-ao), one in which animal–location was excluded (OL-al), and one in which object–location was excluded (OL-ol) (see Figure 1). Thus, events in the open-loop conditions consisted of two overlapping pairs with a common element. The design is an adaptation of the one used by Horner and Burgess (2014) and Horner et al. (2015) in the context of the separated encoding paradigm. We equated the open-loop conditions to the CL condition regarding the number of event elements instead of the number of associations. Previous research yielded similar results when equating the number of associations or event elements (Horner & Burgess, 2014; Joensen et al., 2020).

#### Material

Stimuli consisted of 180 German nouns of three different types—60 animals (all mammals; e.g., *dog*), common objects (e.g., *bucket*), and locations (e.g., *office*). An additional 12 nouns—four animals, common objects, and locations—were used as buffers to avoid primacy effects (primacy buffers). Stimuli were partly taken and adapted from the ones used by Joensen et al. (2020) and translated into German. We used animals instead of famous persons to prevent potential effects of prominence or ignorance of specific persons. From the stimuli, we randomly generated 60 animal–object–location triplets, making up an “event” for each participant. Events were then randomly assigned to the four experimental conditions, resulting in 15 events per condition. In addition, we randomly generated four primacy buffer events, one per condition, which were presented first.

#### Procedure

The experiment was conducted online and implemented using lab.js (Henninger et al., 2020). Data collection was managed by JATOS (Lange et al., 2015). The procedure (see Figure 1) was based on the separated encoding paradigm (Horner & Burgess, 2014; Horner et al., 2015). The experiment consisted of a learning phase, a filler phase, and a test phase. Participants were not made aware of the underlying event structure and were not informed that they would later be tested on the stimuli seen in the learning phase. In the learning phase, events were presented sequentially with two of the constituent elements (i.e., one association) shown per learning trial. There was a minimum of two other event trials between two same event trials. Words were presented to the left or right of the screen centre. The assignment of event element type (e.g., animal) to screen location was randomised. Participants were instructed to imagine the words as elements of a scene as vividly as possible and imagine them interacting in a meaningful manner. Each trial consisted of a 0.5-s fixation cross, followed by the presentation of the word pair for 6 s and a subsequent 1.5-s blank screen. The experimental conditions were randomly distributed across trials. Primacy buffer events were presented first to prevent primacy effects and were not included in the test phase. In the filler phase, participants had to solve randomly generated math problems for 3 min to avoid recency effects.

In the test phase, following a 0.5-s fixation cross, participants were first presented a cue word, which was an event element (e.g., an object) they had seen in the learning phase, in the screen centre for 3 s. Participants then had to give recollection judgements, indicating whether they *remembered* the cue word, merely *knew* that it had been presented in the learning phase or did *not recognise* it. This was done to distinguish between experiences of recollection and familiarity. The instructions for the remember–know distinction closely followed those used by Gardiner (1988), translated into German. Following another 0.5-s fixation cross, participants then conducted a cued recognition forced-choice task. The cue word was displayed in the screen centre, and response alternatives were displayed in a hexagonal array around it. Participants had to choose the target associated with the cue word from the response alternatives. All response alternatives were of the same type (e.g., location) and distractors were randomly drawn from other events. The screen location of the target was randomised. All associations were tested, but only in one direction to avoid testing effects. Thus, there were two possible configurations of cue–target pairs that could be tested for a given event: (a) cue animal and target object, cue object and target location, and cue location and target animal, and (b) cue animal and target location, cue location and target object, and cue object and target location. The direction tested was randomly determined per event, and thus each direction occurred, on average, equally often and randomly distributed across participants. This resulted in three test trials per event. Note that for the open-loop conditions, test trials included one inference trial per event in which the target and cue word were not presented jointly in the learning phase but belong to the same event. While they were not shown as being explicitly related, they could be flexibly related through their overlap with the common event element (for example, if participants learned the associations animal–object and animal–location, they may also imagine a relation between object–location and integrate it into a common memory representation). Thus, for inference trials, a correct response indicates a correct reconstruction of the association that was not shown in the learning phase. The test phase consisted of three blocks, with one association per event tested in each block. Trial order was randomised in each block. Thus, inference trials were intermixed with the other test trials.

#### Data analysis

All analyses were conducted in the R Programming Environment (R Core Team, 2020), and we used the R package *papaja* (Version 0.1.0.9997; Aust & Barth, 2020) for reporting. We used the conventional significance level of α = 5% for all analyses.

##### Exploratory analysis of memory performance

To analyse memory performance, we fit a generalised linear mixed model with a logit link function (see Goldstein, 2011), using the test trial outcomes as a binary dependent variable. Note that the analysis refers to single trials and not aggregated values across trials (see Hoffman & Rovine, 2007). We included random person intercepts and fixed effects for condition, recollection judgement, association^3^, and the interactions. To assess the influence of specific factors, we compared models with isolated effects with a baseline model. For the main effects, the baseline model was the null model that only contained a fixed and a random person intercept. For the two-way interactions, the baseline model was the model with all main effects, and for the three-way interaction, the baseline model was the model with all main effects and two-way interactions. For each effect, we then computed the Bayes factor in favour of an effect $\left({\mathrm{BF}}_{10}\right)$ using Bayesian information criterion (BIC) approximation^4^ (Raftery, 1995; Wagenmakers, 2007). Thus, a Bayes factor >1 is in favour of an effect. A Bayes factor >3 is considered moderate evidence, and a Bayes factor >10 is considered strong evidence for an effect (consequently, Bayes factors <0.33 and <0.1 are considered moderate and strong evidence for the absence of an effect, see Jeffreys, 1961). In addition, we computed the marginal pseudo- $R^{2}$ (Nakagawa et al., 2017), which describes the proportion of variance explained by the fixed effects, for each model and report the change in marginal $R^{2}\;(R_{change}^{2})$ as an indicator of effect size. For the full model, we report both the marginal $R^{2}$ and the conditional $R^{2}$ , which describes the proportion of variance explained by both fixed and random effects. To further investigate effects, we conducted post-hoc pairwise comparisons using the *p* value adjustment by Holm (1979) to account for multiple testing.

Models were fit using the R package *lme4* (Version 1.1-23; Bates et al., 2015). Pseudo- $R^{2}$ were computed using the package *MuMIn* (Version 1.43.17; Barton, 2020) using the delta method. Post-hoc pairwise comparisons were conducted using the package *emmeans* (Version 1.4.7; Lenth, 2020).

##### Analysis of dependency

To model the stochastic dependency of the retrieval of event elements, we employed an IRT (Lord, 1980; Lord & Novick, 1968) approach. Items (i.e., test trials in the cued recognition task, including inference trials) were ordered by condition, event, and cue type. We used a three-parameter logistic model (Birnbaum, 1968) because it allows to control for guessing. It models the probability of person *i* to give a correct response *u* to item *j*, given a latent person trait θ , the item difficulty β , an item-specific discrimination parameter γ , and an item-specific guessing parameter γ :

(1) $$P\left(u_{ij}=1\right)=\gamma_{j}+\left(1-\gamma_{j}\right)\frac{e^{\alpha_{j}(\theta_{i}-\beta_{j})}}{1+e^{\alpha_{j}(\theta_{i}-\beta_{j})}}$$

As events were randomly generated, we fixed discrimination parameters to be equal across trials and set $\alpha_{j}$ to 1. We fixed guessing parameters to the stochastic guessing probability of $\frac{1}{6}$ given six response alternatives. This reduces the model to:

(2) $$P\left(u_{ij}=1\right)=\frac{1}{6}+\frac{5}{6}\frac{e^{{{}_{\theta_{i}-}}_{\beta_{j}}}}{1+\;e^{{{}_{\theta_{i}-}}_{\beta_{j}}}}$$

This model assumes LI of item responses, which means that the latent person trait, reflecting participants’ memory performance, accounts for all inter-item relationships (de Ayala, 2009; Lazarsfeld & Henry, 1968). Consequently, the residual correlations between items should equal zero. This assumption is violated if there are other influences on item responses beyond the latent person trait. Given binding of event elements, there should be additional event-specific effects inducing a dependency of the retrieval of event elements within triplets over and above the dependency induced by the person effect θ . This would violate LI and manifest as nonzero residual correlations of related item pairs. We calculated item residual correlations using the $Q_{3}$ statistic (Yen, 1984). The statistic is calculated for item pairs (*j*, *j*′) in four steps. First, person and item parameters are estimated from the model. Second, the probability of answering items *j* and *j*′ correctly is determined for each person based on the estimated model parameters. Third, the residuals for both items are computed by subtracting the probability of a correct response from the observed response (i.e., 0 or 1) for each person. Finally, the $Q_{3}$ statistic for the item pair is calculated as the correlation of the residuals of both items across persons. Yen (1993) noted that the $Q_{3}$ statistic is negatively biased, with an expected value of $\frac{-1}{I-1}$ given LI, with *I* being the number of items. Thus, we applied a bias correction by subtracting this expected value from all $Q_{3}$ . We defined the stochastic dependency of the retrieval of event elements (*D*) as:

(3) $$D=\frac{1}{K}\sum_{k>k^{\prime }}Q_{3}^{kk^{\prime }}-\frac{1}{L}\sum_{l>l^{\prime }}Q_{3}^{ll^{\prime }}$$

where *kk*′ are item pairs belonging to the same event, *ll*′ are item pairs belonging to different events, *K* is the number of item pairs belonging to the same event, and *L* is the number of item pairs belonging to different events. Given stochastic dependency of the retrieval of event elements, within-event residual correlations should deviate from zero, whereas between-event residual correlations should not. Consequently, *D* should deviate from zero. Note that *D* is rather robust against model misspecification, because this affects both within- and between-event residual correlations. We calculated the dependency estimates for the whole data and for specific recollection judgements (remember, know, and no recognition responses).

Because the sampling distribution of $Q_{3}$ , and consequently the sampling distribution of *D*, is unknown (Chen & Thissen, 1997), we obtained *p* values using parametric bootstrapping. To obtain estimates of event-specific effects to use in the parametric bootstrap, we fit a bifactor model (see Gibbons & Hedeker, 1992; Wainer & Wang, 2000; Supplementary Appendix A). The model extends the unidimensional IRT model in Equation 1 by adding additional latent traits for each event. Thus, items from one event load on one of these additional latent traits, and this trait is thus specific for a given event. These event-specific latent traits capture residual stochastic dependencies within the triplets forming an event. Stochastic dependencies are reflected by the traits’ variances, with higher variances indicating higher stochastic dependencies within events. These variances can be used as indicators of event-specific effects in the parametric bootstrap. We restricted variances of event-specific traits to be equal within conditions, because events were randomly generated. We employed two different approaches. In the first approach, conditional independence depicts the null hypothesis, whereas residual dependencies between items of an event within a condition depict the alternative hypothesis. For this approach, we simulated 1,000 datasets from the unidimensional model in Equation 2. Item parameters were estimated from the data^5^ and person parameters were drawn from a normal distribution with mean zero and variance estimated from the data. We then calculated *D* values for each dataset and recollection judgement, and computed two-tailed *p* values^6^. In the second approach, equal residual dependencies between conditions depict the null hypothesis, whereas differences in residual dependencies between conditions depict the alternative hypothesis. For this approach, we simulated 1,000 datasets per condition from the bifactor model in Equation A2 of Supplementary Appendix A. Item parameters were estimated from the data and person parameters were drawn from a multivariate normal distribution with a zero mean vector and variances estimated from the data. We set variances of all event-specific latent traits equal to the one estimated for the respective focal condition. For obtaining specific estimates for different recollection judgements, we assumed them to be randomly distributed across persons and items, with probability equalling their respective proportion in the data. We then calculated differences between *D* values and computed one-tailed *p* values for the differences^7^. Note that we did not test for differences in dependencies between pairs of conditions if there were negative dependencies in both conditions, because such a comparison is not relevant for the research questions. Further information on the modelling approach is given in Schreiner and Meiser (2022).

We used the R package *mirt* (Version 1.32.1; Chalmers, 2012) and adapted functions from the package *sirt* (Version 3.9-4; Robitzsch, 2020) for the dependency analysis. Simulations were conducted using the package *SimDesign* (Version 2.0.1; Chalmers, 2020). We also report the dependency results obtained using the approach by Horner and Burgess (2013) in Supplementary Appendix B. Results were largely congruent with the ones from the main dependency analysis.

#### Participants

Participants were recruited from the web (social media, mailing lists, forums, blogs, and the online research platform SurveyCircle) and could join a lottery for winning vouchers of a total value of 400€ and receive course credit (SurveyCircle, 2021). A power analysis using simulated data based on data from a pilot study (*n* = 27) for detecting the expected pattern of results with medium effects (differences in event-specific trait variances of 1 according to the statistical procedure; cf. Glas et al., 2000; Wang et al., 2002) between conditions with 80% power (one-tailed testing) yielded a desired sample size of 180 participants. For further information about the power analysis, see Supplementary Appendix C. The experiment was completed by 181 participants. All participants provided online informed consent for their participation and publication of their data. One participant was excluded due to not speaking German fluently. Another participant was excluded due to low accuracy (less than 10%) in the filler task. Another four participants were excluded because they indicated their data should not be used (e.g., due to missing some learning trials). Two additional participants were excluded because they indicated having recently participated in a similar study. Finally, 24 participants were excluded because they interrupted the experiment^8^. This yielded a final sample of 149 participants^9^ (72% female, 1% non-binary, 1% not wanting to disclose their gender; 75% students) with a mean age of 27.0 years (*SD* = 8.5). Data, materials, and analysis scripts for the experiment are provided via the Open Science Framework (OSF; https://osf.io/dt35k/).

### Results

#### Memory performance

**Figure 2.**
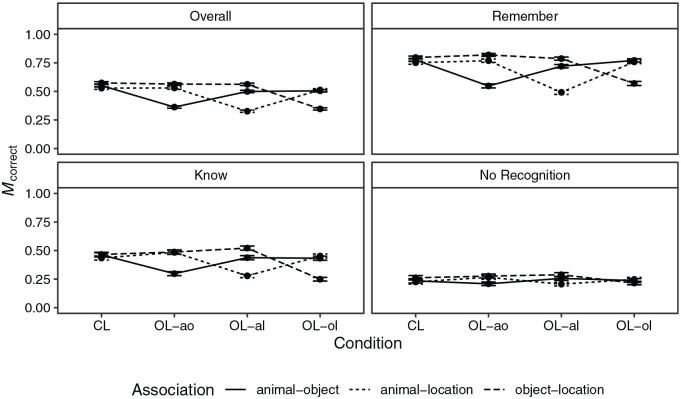
Mean proportion of correct responses by loop condition and association for the whole data (overall) and for subsets of data with specific recollection judgements in Experiment 1. *Note.* CL = closed loop; OL-ao = open loop with association animal–object excluded; OL-al = open loop with association animal–location excluded; OL-ol = open loop with association object–location excluded. Error bars represent ±*SEM*.

Overall, the proportion of correct responses was *M* = 0.49 (*SD* = 0.50). The proportion of correct responses by condition, association, and recollection judgement is shown in Figure 2. Further indices are shown in Table D1 in the Supplementary Appendix. There was strong evidence for main effects of condition ( ${\mathrm{BF}}_{10}$ > 1,000, $R_{change}^{2}$ = .007), recollection judgement ( ${\mathrm{BF}}_{10}$ > 1,000, $R_{change}^{2}$ = .07), and association ( ${\mathrm{BF}}_{10}$ > 1,000, $R_{change}^{2}$ = .001). Post-hoc pairwise comparisons revealed significantly higher performance for remember responses than for know (log-odds ratio [log OR]^10^ = 1.02, *z* = 27.14, *p* < .001) and no recognition responses (log OR = 1.46, *z* = 33.98, *p* < .001), and significantly higher performance for know than for no recognition responses (log OR = 0.44, *z* = 10.79, *p* < .001). There was also strong evidence for a two-way interaction of condition and association ( ${\mathrm{BF}}_{10}$ > 1,000, $R_{change}^{2}$ = .03) which qualified the respective main effects. Post-hoc pairwise comparisons (see Table 1) revealed that memory performance was lowest in conditions in which the respective association was not presented in the learning phase (i.e., inference associations) but did not significantly differ otherwise, except for lower performance for the association animal–object than for object–location in condition OL-al. In condition CL, performance was lower for association animal–location than for object–location but did not significantly differ otherwise. There was strong evidence against two-way interactions of condition and recollection judgement ( ${\mathrm{BF}}_{10}$ < 0.001, $R_{change}^{2}$ = .001) and of recollection judgement and association ( ${\mathrm{BF}}_{10}$ < 0.001, $R_{change}^{2}$ < .001). Finally, there was strong evidence against a three-way interaction ( ${\mathrm{BF}}_{10}$ = 0.02, $R_{change}^{2}$ = .002). The marginal $R^{2}$ of the full model was .11 and the conditional $R^{2}$ was .33.

**Table 1.** Results of the post-hoc pairwise comparisons for the interaction of condition and association regarding memory performance in Experiment 1.

Contrast	Condition	Log OR	*z*	*p*
Animal-object–animal-location	CL	0.13	1.71	.26
Animal-object–object-location	CL	–0.12	–1.63	.26
Animal-location–object-location	CL	–0.25	–3.34	.004
Animal-object–animal-location	OL-ao	–0.90	–12.18	<.001
Animal-object–object-location	OL-ao	–1.06	–14.29	<.001
Animal-location–object-location	OL-ao	–0.17	–2.26	.09
Animal-object–animal-location	OL-al	0.89	12.10	<.001
Animal-object–object-location	OL-al	–0.34	–4.72	<.001
Animal-location–object-location	OL-al	–1.23	–16.63	<.001
Animal-object–animal-location	OL-ol	–0.03	–0.47	.64
Animal-object–object-location	OL-ol	0.84	11.26	<.001
Animal-location–object-location	OL-ol	0.88	11.79	<.001

*Note.* Log OR = log-odds ratio; CL = closed loop; OL-ao = open loop with association animal–object excluded; OL-al = open loop with association animal–location excluded; OL-ol = open loop with association object–location excluded.

#### Dependency

**Figure 3.**
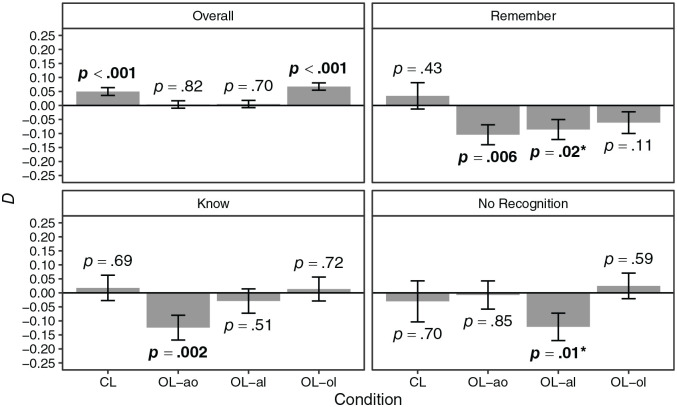
Dependency of the retrieval of event elements by loop condition in Experiment 1 for the whole data (overall) and for subsets of data with specific recollection judgements. *Note.* CL = closed loop; OL-ao = open loop with association animal–object excluded; OL-al = open loop with association animal–location excluded; OL-ol = open loop with association object–location excluded. Error bars represent ±*SE*. The *p* values set in boldface indicate statistical significance at the *p* < .05 level; *p* values marked with an asterisk (*) did no longer indicate statistical significance at the *p* < .05 level after adjusting for multiple comparisons using the *p* value adjustment by Holm (1979); *p* values were obtained using parametric bootstrapping.

Dependencies of the retrieval of event elements are shown in Figure 3. Overall, there was a significant positive dependency in conditions CL and OL-ol but not in conditions OL-ao and OL-al. The dependency in condition CL was significantly larger than the dependency in condition OL-ao ( $D_{diff}$ = 0.05, *p* = .04), although this difference was no longer significant after adjusting for multiple comparisons ( $p_{adj}$ = .14) using the *p* value adjustment by Holm (1979). The dependency in condition CL did not significantly differ from the dependencies in conditions OL-al ( $D_{diff}$ = 0.04, *p* = .06) and OL-ol ( $D_{diff}$ = –0.02, *p* = .13). The dependencies in conditions OL-ao and OL-al did not significantly differ ( $D_{diff}$ = 0.00, *p* = .43) but were significantly smaller than the dependency in condition OL-ol ( $D_{diff}$ = –0.06, *p* = .001 and $D_{diff}$ = –0.06, *p* < .001, respectively). Regarding specific recollection judgements, there were significant negative dependencies for the subset of the data that received remember responses in conditions OL-ao and OL-al, although the latter was no longer significant after adjusting for multiple comparisons ( $p_{adj}$ = .07). The dependency in condition CL was significantly larger than the dependencies in the open-loop conditions ( $D_{diff}$ = 0.14, *p* = .005; $D_{diff}$ = 0.12, *p* = .02; and $D_{diff}$ = 0.10, *p* = .04, respectively). Regarding know responses, there was a significant negative dependency in condition OL-ao. The dependency in condition CL was significantly larger than the dependency in condition OL-ao ( $D_{diff}$ = 0.14, *p* = .02) but did not significantly differ from the dependencies in conditions OL-al ( $D_{diff}$ = 0.05, *p* = .29) and OL-ol ( $D_{diff}$ = 0.00, *p* = .49). The dependency in condition OL-ao was significantly smaller than the dependency in condition OL-ol ( $D_{diff}$ = –0.14, *p* = .03), although this difference was no longer significant after adjusting for multiple comparisons ( $p_{adj}$ = .11). The dependency in condition OL-al did not significantly differ from the dependency in condition OL-ol ( $D_{diff}$ = –0.04, *p* = .27). Regarding no recognition responses, there was a significant negative dependency in condition OL-al, although this dependency was no longer significant after adjusting for multiple comparisons ( $p_{adj}$ = .06). The dependency in condition OL-al was significantly smaller than the dependency in condition OL-ol ( $D_{diff}$ = –0.15, *p* = .03), although this difference was no longer significant after adjusting for multiple comparisons ( $p_{adj}$ = .09). The dependencies in conditions CL and OL-ao did not significantly differ from the dependency in condition OL-ol ( $D_{diff}$ = –0.06, *p* = .25 and $D_{diff}$ = –0.03, *p* = .34, respectively). In summary, there were significant positive dependencies in the CL condition and in the open-loop condition in which the association object–location was excluded. These dependencies were significantly larger than the ones in the other open-loop conditions, in which dependencies did not significantly differ from zero. Dependencies for specific recollection judgements did either not significantly differ from zero or were significantly negative.

### Discussion

In Experiment 1, we replicated the finding that the dependency of the retrieval of event elements is maintained if the encoding of an event occurs in several temporally divided episodes (Horner & Burgess, 2014; Horner et al., 2015; Joensen et al., 2020). Thus, Hypothesis 1, which stated that there is a stochastic dependency of the retrieval of event elements, was supported.

Hypothesis 2, which stated that dependency is only found in the case of recollection, was not supported. Dependency was not only and not consistently found for remember responses. Results regarding specific recollection judgements were inconsistent, and if dependencies reached significance, they were unexpectedly negative. It is also noteworthy that the dependency pattern for the whole data differed considerably from the dependency patterns for specific recollection judgements. This may be because the overall pattern also includes dependencies between item responses associated with different recollection judgements. These are excluded when only considering item responses associated with specific recollection judgements. For example, relationships between event elements may be remembered better for remember than for know responses. This may also extend to item pairs where one item received a remember response and the other received a know response (i.e., despite one item receiving a know response, all relationships are remembered well). However, responses to such item pairs are only considered when using the whole data but not when estimating the dependency for remember or know responses in isolation. The inconsistent findings regarding specific recollection judgements may suggest that the remember–know paradigm in its current implementation is not appropriate for use together with the separated encoding paradigm and the more complex representations studied. The remember–know paradigm (Gardiner, 1988) targets only specific elements. This is appropriate for simpler representations, such as an object with two features. As we closely adapted the paradigm for the current experiment, recollection judgements refer to specific cue words. However, the separated encoding paradigm and the modelling approach operate on the level of associations and whole events. It may be this discrepancy in targeting levels that drives the inconsistent findings regarding specific recollection judgements. Another potential limitation may be participants struggling to understand the remember–know instructions (e.g., see Geraci et al., 2009; Migo et al., 2012), which may limit the validity of the subjective remember–know responses. In addition, differences in information contributing to the dependency estimates for different recollection judgements (i.e., varying number of item responses considered in the computation of the respective estimates) and differences in memory performance associated with different recollection judgements may have limited the equatability of estimates for different recollection judgements, which may have contributed to the unexpected findings. However, we considered these differences in the parametric bootstrap, and thus, the significance patterns of the empirical results should be comparable for different recollection judgements.

Hypothesis 3 stated that dependency is reduced but not eliminated in non-coherent encoding episodes. Dependency was effectively eliminated in conditions OL-ao and OL-al, although dependency in condition CL was not significantly larger than the dependency in condition OL-al and not significantly larger than the dependency in condition OL-ao after adjusting for multiple comparisons. However, this may be due to a power problem. Also note that adjusting *p* values is associated with a loss of statistical power. Nevertheless, the tests against independence clearly support the interpretation that dependency was effectively eliminated in these conditions. In condition OL-ol, however, dependency was maintained and did not significantly differ from the dependency in condition CL. Thus, Hypothesis 3 was not supported. However, this pattern of results supports Hypothesis 4, which stated that dependency varies as a function of the excluded association in non-coherent encoding episodes. Excluding the association object–location in the learning phase did not affect dependency, whereas excluding associations involving the animal did. This was the case even though the pairwise associations did generally not differ regarding memory performance given that they were shown in the learning phase. The pattern of results suggests a hierarchical binding structure in which elements are preferentially bound to the animal. In addition, the results suggest that the encoding episode does not necessarily have to be coherent for dependencies to occur. In Experiment 2, we aimed to replicate these findings and determine whether the observed pattern of results can be attributed to animacy influencing the binding of event elements.

## Experiment 2

Human memory functioning may be a product of selective pressure on our ancestors (Nairne et al., 2007, 2008). In this context, animacy may be an especially important survival-related factor influencing human cognition (Nairne et al., 2013, 2017). For example, words with an animate referent are retrieved more likely than words with an inanimate referent, a phenomenon termed the animacy effect (e.g., Li et al., 2016; Nairne et al., 2013; VanArsdall et al., 2015). Such an animacy effect has been found for several types of tasks such as free recall (Bonin et al., 2015; Li et al., 2016; Madan, 2021; Nairne et al., 2013; Popp & Serra, 2016), cued recall (DeYoung & Serra, 2021; Laurino & Kaczer, 2019; VanArsdall et al., 2015; but note Kazanas et al., 2020; Popp & Serra, 2016, who found reduced performance for animate referents in cued recall tasks), free recognition (Bonin et al., 2014; see also VanArsdall et al., 2013), and judgements of learning (DeYoung & Serra, 2021; Li et al., 2016). Animate entities are defined as being living things which are capable of independent movement and can change direction without warning (Bonin et al., 2015). The animal event elements in Experiment 1 meet this definition. Given that the results of Experiment 1 suggest that elements are preferentially bound to the animal and the importance of animacy in human cognition (Nairne et al., 2013, 2017), it may be that animacy affects not only the retrieval but also the binding of event elements. For example, animacy may qualify the referent word to be an initiator of action, thus qualifying it to be the grammatical subject in sentences describing events, whereas inanimate objects or locations are grammatical objects.

In Experiment 2, we aimed to investigate whether animacy was responsible for the effect found in Experiment 1. To this end, we constructed events that either include an animate element, as was the case in Experiment 1, or do not include an animate element. If animacy is responsible for the effect in Experiment 1, the dependency of the retrieval of event elements should vary as a function of the excluded association in non-coherent encoding episodes if events include an animate element (Hypothesis 5a). Specifically, for these events, the pattern of results of Experiment 1 should be replicated. However, the dependency of the retrieval of event elements should not vary as a function of the excluded association in non-coherent encoding episodes if events do not include an animate element (Hypothesis 5b). We decided not to further investigate dependency for different recollection judgements, but instead focus on the main research questions of how the binding of event elements in episodic memory is structured and whether animacy influences binding. The experiment was preregistered at https://osf.io/m2fjv.

### Method

#### Design

Half of the events included an animate entity and the other half did not, leading to a 2 (animacy condition: animacy vs. non-animacy) × 4 (loop condition: CL and three open loops) within-subjects design. For the animacy condition, loop conditions were identical to those of Experiment 1. In the open-loop non-animacy conditions, the association means of transportation–tool (OL- $o_{tr}o_{to}$ ), means of transportation–location (OL- $o_{tr}$ l), or means of tool–location (OL- $o_{to}$ l) was excluded from presentation (see Figure 1).

#### Material

Stimuli consisted of 192 German nouns, partly taken from Experiment 1, of four different types—32 animals (all mammals), 48 objects representing means of transportation (e.g., *bicycle*), 48 objects representing tools (e.g., *hammer*), and 64 locations. An additional 24 nouns—four animals, six means of transportation, six tools, and eight locations—were used as primacy buffers. From the stimuli, we randomly created 64 triplets, making up an “event” for each participant. Half of the events consisted of an animal, an object (balanced as to whether being a means of transportation or a tool), and a location (animacy condition). The other half consisted of two objects (one means of transportation and one tool) and a location (non-animacy condition). Events were then randomly assigned to the eight experimental conditions, resulting in eight events per condition.^11^ In addition, we randomly generated eight primacy buffer events, one per condition, which were presented first.

#### Procedure

The procedure was identical to the one of Experiment 1 with the following exceptions: For each participant, stimuli were kept separate for the animacy and non-animacy conditions to keep the number of possible distractors in the test phase equal between different types of elements. To achieve this, one-third of the means of transportation and the tools stimuli were initially randomly assigned to the animacy condition, while the remaining ones were used for the non-animacy condition. In addition, we did not collect recollection judgements in this experiment. Thus, a test trial only consisted of a 0.5-s fixation cross, followed by a 3-s cue presentation, followed by another 0.5-s fixation cross, followed by the cued recognition task.

#### Data analysis

Data analysis was identical to the one conducted in Experiment 1 except that we did not consider recollection judgements in this experiment. For the exploratory analysis of memory performance, we included loop condition, animacy condition, association, and the interactions as fixed effects in the generalised linear mixed model. We coerced the associations animal–object and means of transportation–tool, animal–location and means of transportation–location, and object–location and tool–location into a common factor level, respectively. We also coerced loop conditions OL-ao and OL- $o_{tr}o_{to}$ , OL-al and OL- $o_{tr}$ l, and OL-ol and OL- $o_{to}$ l into a common factor level, respectively.^12^ For the dependency analysis, *p* values were again obtained using parametric bootstrapping.

#### Participants

Participants were recruited from the web and could receive course credit or a monetary compensation of 3€ and join a lottery for winning vouchers of a total value of 100€. A power analysis using simulated data based on Experiment 1 for detecting the expected pattern of results with small to medium effects (differences in event-specific trait variances of 0.75; cf. Glas et al., 2000; Wang et al., 2002) between conditions with 80% power (one-tailed testing) yielded a desired sample size of 210 participants. Given the observed exclusion rate in Experiment 1, we decided to increase the desired sample size by 20% and thus collected data of 252 participants. All participants provided online informed consent for their participation and publication of their data. Two participants were excluded due to not speaking German fluently. Another two participants were excluded due to low accuracy (less than 10%) in the filler task. Another three participants were excluded because they indicated their data should not be used (e.g., due to distractions). Two additional participants were excluded because they indicated having recently participated in a similar study. Finally, 30 participants were excluded because they interrupted the experiment. This yielded a final sample of 213 participants (73% female, 0.5% non-binary, 1% not wanting to disclose their gender; 80% students) with a mean age of 27.3 years (*SD* = 9.5). Data, materials, and analysis scripts for the experiment are provided via the OSF (https://osf.io/dt35k/).

### Results

#### Memory performance

**Figure 4.**
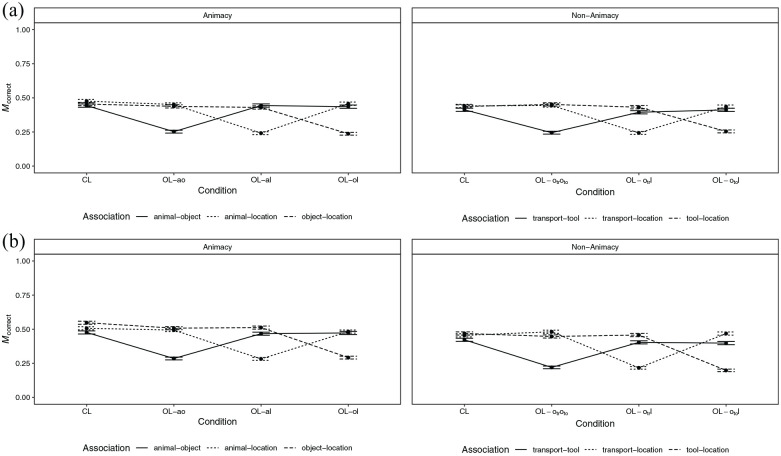
Mean proportion of correct responses by animacy condition, loop condition, and association in (a) Experiment 2 and (b) Experiment 3. *Note.* CL = closed loop; OL-ao = open loop with association animal–object excluded; OL-al = open loop with association animal–location excluded; OL-ol = open loop with association object–location excluded; OL- $o_{tr}o_{to}$ = open loop with association means of transportation–tool excluded; OL- $o_{\mathrm{tr}}$ l = open loop with association means of transportation–location excluded; OL- $o_{to}$ l = open loop with association tool–location excluded; transport = means of transportation. Error bars represent ±*SEM*.

Overall, the proportion of correct responses was *M* = 0.40 (*SD* = 0.49) in the animacy condition and *M* = 0.38 (*SD* = 0.49) in the non-animacy condition. The proportion of correct responses by loop condition, animacy condition, and association is shown in Figure 4a. Further indices are shown in Table D2 in the Supplementary Appendix. There was strong evidence for a main effect of loop condition ( ${\mathrm{BF}}_{10}$ > 1,000, $R_{change}^{2}$ = .005) but weak evidence against a main effect of animacy condition ( ${\mathrm{BF}}_{10}$ = 0.59, $R_{change}^{2}$ < .001) and strong evidence against a main effect of association ( ${\mathrm{BF}}_{10}$ = 0.02, $R_{change}^{2}$ < .001). There was strong evidence for a two-way interaction of loop condition and association ( ${\mathrm{BF}}_{10}$ > 1,000, $R_{change}^{2}$ = .04) which qualified the main effect of loop condition. Post-hoc pairwise comparisons (see Table 2) revealed that memory performance was lowest in conditions in which the respective association was not presented in the learning phase (i.e., inference associations) but did not significantly differ otherwise. In condition CL, performance was lower for association animal–object/transport–tool than for object–location/tool–location but did not significantly differ otherwise. There was strong evidence against two-way interactions of loop condition and animacy condition ( ${\mathrm{BF}}_{10}$ < 0.001, $R_{change}^{2}$ < .001) and of animacy condition and association ( ${\mathrm{BF}}_{10}$ = 0.003, $R_{change}^{2}$ < .001). Finally, there was strong evidence against a three-way interaction ( ${\mathrm{BF}}_{10}$ < 0.001, $R_{change}^{2}$ < .001). The marginal $R^{2}$ of the full model was .04 and the conditional $R^{2}$ was .28.

**Table 2.** Results of the post-hoc pairwise comparisons for the interaction of loop condition and association regarding memory performance in Experiment 2.

Contrast	Loop condition	Log OR	*z*	*p*
Animal-object/transport-tool–animal-location/transport-location	CL	–0.16	–2.89	.02
Animal-object/transport-tool–object-location/tool-location	CL	–0.09	–1.71	.35
Animal-location/transport-location–object-location/tool-location	CL	0.07	1.19	.71
Animal-object/transport-tool–animal-location/transport-location	OL-ao/ $o_{\mathrm{tr}}\;o_{\mathrm{to}}$ otroto	–1.17	–19.48	<.001
Animal-object/transport-tool–object-location/tool-location	OL-ao/ $o_{\mathrm{tr}}\;o_{\mathrm{to}}$ otroto	–1.15	–19.22	<.001
Animal-location/transport-location–object-location/tool-location	OL-ao/ $o_{\mathrm{tr}}\;o_{\mathrm{to}}$ otroto	0.02	0.28	.78
Animal-object/transport-tool–animal-location/transport-location	OL-al/ $o_{tr}$ l	1.06	17.52	<.001
Animal-object/transport-tool–object-location/tool-location	OL-al/ $o_{tr}$ l	–0.06	–1.11	.71
Animal-location/transport-location–object-location/tool-location	OL-al/ $o_{tr}$ l	–1.12	–18.58	<.001
Animal-object/transport-tool–animal-location/transport-location	OL-ol/ $o_{to}$ l	–0.12	–2.15	.16
Animal-object/transport-tool–object-location/tool-location	OL-ol/ $o_{to}$ l	1.06	17.61	<.001
Animal-location/transport-location–object-location/tool-location	OL-ol/ $o_{to}$ l	1.18	19.64	<.001

*Note.* Log OR = log-odds ratio; CL = closed loop; OL-ao = open loop with association animal–object excluded; OL-al = open loop with association animal–location excluded; OL-ol = open loop with association object–location excluded; OL-otroto $o_{tr}o_{to}$ = open loop with association means of transportation–tool excluded; OL-otr $o_{tr}$ l = open loop with association means of transportation–location excluded; OL-oto $o_{\mathrm{to}}$ l = open loop with association tool–location excluded; transport = means of transportation. Associations and loop conditions separated by a slash (/) were treated as one factor level, respectively.

#### Dependency

**Figure 5.**
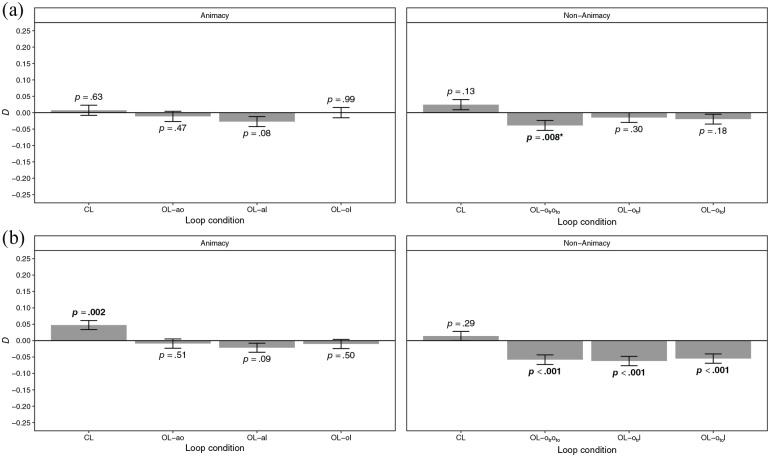
Dependency of the retrieval of event elements in the animacy and non-animacy conditions of (a) Experiment 2 and (b) Experiment 3 by loop condition. *Note.* CL = closed loop; OL-ao = open loop with association animal–object excluded; OL-al = open loop with association animal–location excluded; OL-ol = open loop with association object–location excluded; OL- $o_{tr}o_{to}$ = open loop with association means of transportation–tool excluded; OL- $o_{tr}$ l = open loop with association means of transportation–location excluded; OL- $o_{to}$ l = open loop with association tool–location excluded. Error bars represent ±*SE*. The *p* values set in boldface indicate statistical significance at the *p* < .05 level; *p* values marked with an asterisk (*) did no longer indicate statistical significance at the *p* < .05 level after adjusting for multiple comparisons using the *p* value adjustment by Holm (1979); *p* values were obtained using parametric bootstrapping.

Dependencies of the retrieval of event elements are shown in Figure 5a. There were no significant dependencies in all conditions except for a negative dependency in loop condition OL- $o_{\mathrm{tr}}o_{to}$ in the non-animacy condition, which was no longer significant after adjusting for multiple comparisons ( $p_{adj}$ = .06) using the *p* value adjustment by Holm (1979). The dependency in loop condition CL in the non-animacy condition was significantly larger than the dependency in loop condition OL- $o_{\mathrm{tr}}o_{\mathrm{to}}$ ( $D_{diff}$ = 0.06, *p* = .02). All other relevant differences were non-significant (*p* ⩾ .10).

### Discussion

In Experiment 2, we did not find evidence for substantial dependencies of the retrieval of event elements. We could neither replicate the positive dependency in condition CL nor the positive dependency in condition OL-ol from Experiment 1. Thus, the results cannot properly distinguish between an integrated and a hierarchical binding structure. As the pattern of results was similar for the animacy and non-animacy condition, there was also no evidence for a special role of animacy. Contrary to Experiment 1, in which events consisted of an animal, an object, and a location, the event structure in Experiment 2 was not always the same. Due to the full within-subjects design and the inclusion of a non-animacy condition, events could either consist of an animal, an object, and a location, or of two objects and a location. As these different event structures were presented in randomly alternating sequence, this may have more strongly concealed the underlying event structure. Thus, participants may not have been as aware of the event structures as in Experiment 1, preventing them from forming abstract representations of event structures, which may have caused them to use different encoding strategies (cf. N. W. Morton et al., 2020; see also Kumaran & Ludwig, 2013). For example, N. W. Morton et al. (2020) suggested that the formation of abstract event structures facilitates binding and particularly supports inference. The results do not preclude a hierarchical binding structure with animal as the critical element, but the varying event structures due to the full within-subjects design may have prevented the formation of coherent memory structures. This could be an additional moderator which requires further examination. In addition, the number of events per condition was reduced from 15 in Experiment 1 to 8 in Experiment 2. Thus, the condition-specific results are based on less information than in Experiment 1. To make the experimental design more similar to Experiment 1 and to rule out potential influences of different degrees of event structure awareness caused by varying event structures, in Experiment 3 we varied animacy as a between-subjects instead of a within-subjects factor and increased the number of events per condition back to 15.

## Experiment 3

In Experiment 3, we again aimed to investigate whether animacy was responsible for the effect found in Experiment 1, while avoiding potential confounds which may have been present in Experiment 2. Thus, we varied animacy as a between-subjects factor and used the same number of events per condition as in Experiment 1. The experiment was preregistered at https://osf.io/vprxd.

### Method

#### Design

The experimental design was identical to the one of Experiment 2 with the exception that animacy was manipulated as a between-subjects instead of a within-subjects factor. This resulted in a 2 (animacy condition: animacy vs. non-animacy) × 4 (loop condition: CL and three open loops) mixed design.

#### Material

Stimuli consisted of 240 German nouns, partly taken from Experiments 1 and 2, of four different types—60 animals (all mammals), 60 objects representing means of transportation, 60 objects representing tools, and 60 locations. An additional 16 nouns—four animals, means of transportation, tools, and locations—were used as primacy buffers. From the stimuli, we randomly created 60 triplets, making up an “event” for each participant. In the animacy condition, events consisted of an animal, an object (balanced as to whether being a means of transportation or a tool), and a location. In the non-animacy condition, events consisted of two objects (one means of transportation and one tool) and a location. Events were then randomly assigned to the four within-subjects conditions, resulting in 15 events per loop condition. In addition, we randomly generated four primacy buffer events, one per loop condition, which were presented first.

#### Procedure

The procedure was identical to the one of Experiment 2. In the animacy condition, for each participant, 30 means of transportation and 30 tools were randomly drawn from the respective lists to serve as object elements.

#### Data analysis

Data analysis was identical to the one conducted in Experiment 2 except that we used animacy condition as a between-subjects factor in the exploratory analysis of memory performance and fit separate models to the data of each animacy condition for the dependency analysis. For the dependency analysis, *p* values were again obtained using parametric bootstrapping.

#### Participants

Participants were recruited from the web and could join a lottery for winning vouchers of a total value of 450€ and earn course credit. A power analysis using simulated data based on Experiment 1 for detecting the expected pattern of results with medium effects (differences in event-specific trait variances of 1; cf. Glas et al., 2000; Wang et al., 2002) between conditions with 80% power (one-tailed testing) yielded a desired sample size of 260 participants (130 per between-subjects condition). Given the observed exclusion rate in Experiment 2, we decided to increase the desired sample size by 15%, and thus collected data of 299 participants (152 in the animacy condition and 147 in the non-animacy condition). All participants provided online informed consent for their participation and publication of their data. Five participants were excluded due to not speaking German fluently. Another four participants were excluded due to low accuracy (less than 10%) in the filler task. Another 10 participants were excluded because they indicated their data should not be used (e.g., due to technical problems or distractions). Four additional participants were excluded because they indicated having recently participated in a similar study. Finally, 23 participants were excluded because they interrupted the experiment. This yielded a final sample of 253 participants (131 in the animacy condition and 122 in the non-animacy condition; 75% female, 1.6% non-binary, 1% not wanting to disclose their gender; 81% students) with a mean age of 27.2 years (*SD* = 9.1). Data, materials, and analysis scripts for the experiment are provided via the OSF (https://osf.io/dt35k/).

### Results

#### Memory performance

Overall, the proportion of correct responses was *M* = 0.44 (*SD* = 0.50) in the animacy condition and *M* = 0.39 (*SD* = 0.49) in the non-animacy condition. The proportion of correct responses by loop condition, animacy condition, and association is shown in Figure 4b. Further indices are shown in Table D3 in the Supplementary Appendix. There was strong evidence for a main effect of loop condition ( ${\mathrm{BF}}_{10}$ > 1,000, $R_{change}^{2}$ = .007) and of association ( ${\mathrm{BF}}_{10}$ > 1,000, $R_{change}^{2}$ = .001), but strong evidence against a main effect of animacy condition ( ${\mathrm{BF}}_{10}$ = 0.04, $R_{change}^{2}$ = .004). There was strong evidence for a two-way interaction of loop condition and association ( ${\mathrm{BF}}_{10}$ > 1,000, $R_{change}^{2}$ = .05) which qualified the respective main effects. Post-hoc pairwise comparisons (see Table 3) revealed that memory performance was lowest in conditions in which the respective association was not presented in the learning phase (i.e., inference associations). In addition, performance was lower for association animal–object/transport–tool than for object–location/tool–location in condition OL-al and lower for association animal–object/transport–tool than for animal–location/transport–location in condition OL-ol. In condition CL, performance was highest for association object–location/tool–location and lowest for association animal–object/transport–tool. Other comparisons were not significant. There was strong evidence against two-way interactions of loop condition and animacy condition ( ${\mathrm{BF}}_{10}$ < 0.001, $R_{change}^{2}$ < .001) and of animacy condition and association ( ${\mathrm{BF}}_{10}$ = 0.02, $R_{change}^{2}$ < .001). Finally, there was strong evidence against a three-way interaction ( ${\mathrm{BF}}_{10}$ < 0.001, $R_{change}^{2}$ = .001). The marginal $R^{2}$ of the full model was .06 and the conditional $R^{2}$ was .27.

**Table 3.** Results of the post-hoc pairwise comparisons for the interaction of loop condition and association regarding memory performance in Experiment 3.

Contrast	Loop condition	Log OR	*z*	*p*
Animal-object/transport-tool–animal-location/transport-location	CL	–0.16	–3.12	.005
Animal-object/transport-tool–object-location/tool-location	CL	–0.30	–5.82	<.001
Animal-location/transport-location–object-location/tool-location	CL	–0.14	–2.71	.01
Animal-object/transport-tool–animal-location/transport-location	OL-ao/ $o_{tr}\;o_{to}$	–1.30	–23.41	<.001
Animal-object/transport-tool–object-location/tool-location	OL-ao/ $o_{tr}\;o_{to}$	–1.26	–22.54	<.001
Animal-location/transport-location–object-location/tool-location	OL-ao/ $o_{tr}\;o_{to}$	0.05	0.92	.36
Animal-object/transport-tool–animal-location/transport-location	OL-al/ $o_{tr}$ l	1.06	18.98	<.001
Animal-object/transport-tool–object-location/tool-location	OL-al/ $o_{tr}$ l	–0.25	–4.83	<.001
Animal-location/transport-location–object-location/tool-location	OL-al/ $o_{tr}$ l	–1.31	–23.51	<.001
Animal-object/transport-tool–animal-location/transport-location	OL-ol/ $o_{to}$ l	–0.20	–3.88	<.001
Animal-object/transport-tool–object-location/tool-location	OL-ol/ $o_{to}$ l	1.10	19.50	<.001
Animal-location/transport-location–object-location/tool-location	OL-ol/ $o_{to}$ l	1.30	23.13	<.001

*Note.* Log OR = log-odds ratio; CL = closed loop; OL-ao = open loop with association animal–object excluded; OL-al = open loop with association animal–location excluded; OL-ol = open loop with association object–location excluded; OL-otroto $o_{tr}o_{to}$ = open loop with association means of transportation–tool excluded; OL- $o_{tr}$ l = open loop with association means of transportation–location excluded; OL- $o_{to}$ l = open loop with association tool–location excluded; transport = means of transportation. Associations and loop conditions separated by a slash (/) were treated as one factor level, respectively.

#### Dependency

Dependencies of the retrieval of event elements are shown in Figure 5b. In the animacy condition, there was a significant positive dependency in condition CL but no significant dependencies in the open-loop conditions. The dependency in condition CL was significantly larger than the dependencies in the open-loop conditions ( $D_{diff}$ = 0.06, *p* = .007; $D_{diff}$ = 0.07, *p* = .003; and $D_{diff}$ = 0.06, *p* = .01, respectively). In the non-animacy condition, there was no significant dependency in condition CL but significant negative dependencies in the open-loop conditions. The dependency in condition CL was significantly larger than the dependencies in the open-loop conditions ( $D_{diff}$ = 0.07, *p* = .001; $D_{diff}$ = 0.08, *p* < .001; and $D_{diff}$ = 0.07, *p* = .002, respectively).

### Discussion

In Experiment 3, we could replicate the positive dependency in condition CL in the animacy condition, thus supporting Hypothesis 1, which stated that there is a stochastic dependency of the retrieval of event elements. Dependencies were close to zero in the open-loop conditions in the animacy condition and negative in the open-loop conditions in the non-animacy condition. Thus, Hypothesis 3, which stated that dependency is reduced but not eliminated in non-coherent encoding episodes, was not supported. The negative dependencies in the non-animacy condition indicate that successful retrieval of one event element is associated with a decreased likelihood of retrieving another event element of the same event. One explanation for this may be that learning trials were encoded as distinct overlapping events. Zotow et al. (2020) found negative dependencies in such a case and suggested that they may be due to pattern separation processes in the hippocampus driving individual event representations apart. Another explanation may be retrieval-induced forgetting (Anderson et al., 1994). The selective retrieval of an event element (e.g., tool when cued by location) may inhibit the non-tested element (e.g., means of transportation), which is then retrieved less likely in the subsequent test trial in which it is the target (cf. Horner & Burgess, 2013). This may have particularly occurred in the non-animacy condition, because it contained two element types, means of transportation and tools, for which object could be considered a superordinate category. Thus, means of transportation and tools may be considered to be more similar semantic categories than, for example, animal and object, which may have facilitated retrieval-induced forgetting (cf. Hicks & Starns, 2004).

We could not replicate the positive dependency in condition OL-ol which was observed in Experiment 1. Thus, Hypothesis 4, which stated that dependency varies as a function of the excluded association in non-coherent encoding episodes, was not supported in Experiment 3, and the pattern of results is in favour of an integrated binding structure. Hypotheses 5a and 5b stated that dependency varies as a function of the excluded association in non-coherent encoding episodes if events include an animate element, but does not vary if evens do not include an animate element. While dependencies in the open-loop conditions in the non-animacy condition were very similar, thus supporting Hypothesis 5b, they were negative. In addition, dependencies did not vary across the open-loop conditions in the animacy condition. Thus, Hypothesis 5a was not supported. However, the results still suggest that animacy influences the binding of event elements. Rather than characterising the element to which other event elements are preferentially bound, as implied by Hypotheses 5a and 5b, the results suggest that animacy facilitates the binding of event elements if the encoding episode is coherent. In the absence of animacy, this integration seems to be less successful, as indicated by the non-significant dependency in condition CL in the non-animacy condition. In addition, if animacy is not present in an event and the event is encoded as temporally divided episodes, the different learning trials may be encoded as distinct events.

## Effect of presentation order regarding animacy

We only observed positive stochastic dependencies of the retrieval of event elements for events that include an animate element (i.e., an animal in the current experiments) across experiments. This may be because animacy provides a potential agent in an event, which may facilitate the formation of coherent memory representations. Consequently, dependencies may be larger for events for which an association involving an animal (i.e., animal–object or animal–location) was presented first compared with events for which an association not involving an animal (i.e., object–location) was presented first. To examine whether this interpretation may be valid, we conducted an exploratory post hoc analysis of presentation order regarding animacy.

We computed dependencies separately for events for which an association involving an animal was presented first and for events for which it was not by declaring respective responses as missing values and then fitting separate models for the two cases. For this analysis, we only considered the animacy conditions, excluding condition OL-ol because in this condition only associations involving an animal were presented. For the bootstrap, we used estimates from the main models but declared some event responses as missing values based on the proportion of events for which an association involving an animal was presented first or not first in each experiment and considered condition.

The results are shown in Table 4. Of the conditions that yielded significant positive dependencies in the main dependency analyses (condition CL in Experiments 1 and 3)^13^, we only found significant dependencies for events for which an association involving an animal was presented first, but not for events for which an association not involving an animal was presented first. This is in favour of the interpretation that the presence of an animate element in an event facilitates the formation of coherent memory representations by providing a potential agent.

**Table 4.** Dependency for events for which an association involving an animate element was presented first or not first per experiment and condition.

Experiment	Condition	Animate element first	*D*	*p*
1	CL	Yes	0.06	.002
1	CL	No	0.03	.26
1	OL-ao	Yes	0.00	.83
1	OL-ao	No	0.00	.97
1	OL-al	Yes	0.01	.53
1	OL-al	No	0.01	.75
2	CL	Yes	–0.01	.56
2	CL	No	0.03	.28
2	OL-ao	Yes	–0.02	.51
2	OL-ao	No	–0.01	.68
2	OL-al	Yes	–0.05	.02
2	OL-al	No	0.00	.97
3	CL	Yes	0.07	<.001
3	CL	No	–0.01	.90
3	OL-ao	Yes	–0.01	.47
3	OL-ao	No	0.00	.81
3	OL-al	Yes	–0.04	.06
3	OL-al	No	0.00	.81

*Note.* CL = closed loop; OL-ao = open loop with association animal–object excluded; OL-al = open loop with association animal–location excluded. Only animacy conditions excluding condition OL-ol were considered.

## General discussion

The purpose of this research was to determine whether event elements in episodic memory are bound in an integrated or a hierarchical manner and, based on the results of the first experiment, investigate whether the presence of animacy in an event influences the binding of its constituent elements, while introducing a new approach for modelling dependencies of the retrieval of event elements in episodic memory. The results of this research cannot clearly distinguish between an integrated and a hierarchical binding structure. However, they provide evidence that animacy influences the binding of event elements. In addition, they hint at a role of awareness regarding the structure of event elements in the binding of event elements.

In two out of three experiments, we found a positive stochastic dependency of the retrieval of event elements in coherent encoding episodes (closed-loop structures) if one of the event elements was animate. This is consistent with the previous literature (Horner & Burgess, 2014; Horner et al., 2015; James et al., 2020; Ngo et al., 2019) and supports Hypothesis 1. It indicates that event elements are bound together even if an event is experienced as several temporally divided encoding episodes. We did not find this effect in Experiment 2, in which events could take different structures for the same participant. In addition, encoding episodes referring to events with different structures were presented in randomly alternating sequence. Thus, the underlying event structure, while being implicit in all experiments, was likely harder for participants to determine in Experiment 2. This reduced awareness regarding the structure of event elements may have prevented participants from forming abstract representations of event structures (cf. N. W. Morton et al., 2020; see also Kumaran & Ludwig, 2013) and may have caused them to use different encoding strategies in Experiment 2 compared with Experiments 1 and 3. The results thus hint at a moderating influence of event structure awareness on the binding of event elements, which may be influenced by perceived task demands. This is consistent with relational memory theory (Cohen & Eichenbaum, 1993; Eichenbaum, 1999), which suggests that task demands affect the binding of event elements. Interestingly, Horner and Burgess (2014) and Horner et al. (2015) also varied event structures and still found a significant dependency of the retrieval of event elements. In their experiments, each element type appeared equally often. This was not the case in our Experiment 2, in which there were fewer animals than means of transportation and tools (the two object categories used) and fewer means of transportation and tools than locations. In addition, their experiments encompassed fewer events than ours (36 events compared with 64 events in Experiment 2), which may have reduced participants’ memory load compared with our experiments. These factors may have contributed to an increased awareness regarding event structures in the experiments by Horner and Burgess (2014) and Horner et al. (2015) compared with Experiment 2.

We also investigated how the binding of event elements differs regarding different recollection judgements. Whereas past research has only observed stochastic dependencies of the retrieval of event elements for remember responses but not for know responses (Boywitt & Meiser, 2012a, 2012b; Meiser & Bröder, 2002; Meiser et al., 2008; Starns & Hicks, 2005), the present study did not find a consistent pattern across different recollection judgements, and dependencies were mostly unexpectedly negative. There is thus no support for Hypothesis 2. However, the remember–know paradigm (Gardiner, 1988) was usually used in the context of item-based representations and targets only specific cue elements. In the context of more complex event-based representations (cf. Andermane et al., 2021; Joensen et al., 2020), which were the focus of the current research, this leads to a discrepancy in targeting levels between the remember–know paradigm and the experimental paradigm and modelling approach, because the latter operate on the level of associations and whole events. This discrepancy may explain the inconsistent findings regarding recollection judgements. The results suggest that the remember–know paradigm may not be readily transferable to more complex representations, at least not in the form of our adaptation of the paradigm.

Regarding non-coherent encoding episodes (open-loop structures), dependencies were either close to zero or not reduced compared with coherent encoding episodes, at least if events contained an animate element. The results do not support Hypothesis 3 but are partly consistent with previous research, which found dependencies only in coherent but not in non-coherent encoding episodes (Horner & Burgess, 2014; Horner et al., 2015; Joensen et al., 2020). Indeed, non-coherent encoding episodes seem to generally disrupt the formation of coherent memory representations, as indicated by the absence of dependencies, or facilitate the formation of pairwise bindings, as opposed to higher level binding structures, which then exhibit mutual inhibition or suppression, as may be indicated by the negative dependencies in conditions in which events did not contain an animate element. Potential mechanisms behind negative dependencies may include pattern separation processes in the hippocampus, which drive individual representations apart (cf. Zotow et al., 2020), or retrieval-induced forgetting (Anderson et al., 1994).

The finding that dependency varied across the non-coherent encoding conditions in Experiment 1, with higher dependency if the association object–location than the associations animal–object or animal–location were excluded from the learning phase, supports Hypothesis 4 and suggests a hierarchical binding structure in which event elements are preferentially bound to the animal. This contradicts previous accounts and interpretations of the binding of event elements as being integrative, such as accounts advocating that event elements are bound into a single coherent event representation or engram (Damasio, 1989; Horner & Burgess, 2014; Horner et al., 2015; Joensen et al., 2020; Marr, 1971; Moll & Miikkulainen, 1997; Tulving, 1983) and the integrative encoding hypothesis (Shohamy & Wagner, 2008; Zeithamova et al., 2012). Rather, the finding is consistent with accounts considering asymmetrical binding such as the ensemble encoding account (Cai et al., 2016), relational memory theory (Cohen & Eichenbaum, 1993; Eichenbaum, 1999), the TEC (Hommel et al., 2001), and the Span–Cospan model of episodic memory (Healy & Caudell, 2019). However, in Experiment 3, the dependency in the non-coherent encoding condition with association object–location being excluded could not be replicated. Dependencies in the non-coherent encoding episodes were all close to zero. This is in favour of an integrated binding structure and thus consistent with integrative binding accounts (Damasio, 1989; Horner & Burgess, 2014; Horner et al., 2015; Joensen et al., 2020; Marr, 1971; Moll & Miikkulainen, 1997; Shohamy & Wagner, 2008; Tulving, 1983; Zeithamova et al., 2012). The results of Experiment 2 are not diagnostic for distinguishing between an integrated and a hierarchical binding structure because even the established finding of a dependency in the coherent encoding condition was not replicated. Taken together, evidence for Hypothesis 4 is ambiguous, and thus the results do not clearly distinguish between an integrated and a hierarchical binding structure. It may well be the case that both integrated and hierarchical binding structures are possible, with the binding structure formed determined by several moderators. James et al. (2020) already identified the modality of stimulus presentation and the dimensionality of presentation modality as potential moderators of the binding of event elements in the context of the separated encoding paradigm (Horner & Burgess, 2014; Horner et al., 2015).

Another moderator may be animacy (e.g., see Bonin et al., 2015; Nairne et al., 2013, 2017). In the current research, positive stochastic dependencies have only been observed for events that include an animate element. However, in Experiment 3, in which events with an animate element and events without any animate element were directly contrasted, dependency did not vary across the non-coherent encoding conditions and was even negative for events without an animate element. These results do not support Hypotheses 5a and 5b but still suggest an influence of animacy. Rather than characterising the prominent event element in a hierarchical binding structure, animacy seems to facilitate the binding of event elements per se, at least in the case of coherent encoding episodes. Although dependencies could also result from processes occurring during retrieval rather than encoding (e.g., Kumaran & McClelland, 2012), we would argue that *differences* in the stochastic dependencies of the retrieval of event elements between animacy conditions imply that there are also differences in the internal representations of the events between the conditions. We prefer to interpret these representation differences in terms of “binding” because this provides a coherent interpretation, but other theoretical ideas may also be viable. The negative dependencies (i.e., successful retrieval of an event element being associated with reduced probability to retrieve another event element) found for events without an animate element may be due to retrieval-induced forgetting (Anderson et al., 1994). Another explanation may be that the temporally divided encoding episodes are represented as distinct overlapping events, thus consisting of pairwise bindings. Zotow et al. (2020) found negative dependencies for partially overlapping events and attributed these to pattern separation processes in the hippocampus which drive representations apart, decreasing their similarity. One could argue that negative dependencies may also occur due to between-event binding of event elements, for example, due to the prevalence of systematic conjunction errors (e.g., Reinitz et al., 1992). This was not the case in the experiments because mean between-event residual correlations were very close to zero in all conditions and experiments. Animacy may provide structure to an event by providing a potential agent. This may enable encoding strategies such as representing the event as a sentence, with the agent as the grammatical subject. In the absence of a prominent agent, events may not be as clearly structured and such encoding strategies not as easily applicable. Consequently, people may resort to pairwise bindings (see Cai et al., 2016; Cohen & Eichenbaum, 1993; Eichenbaum, 1999). In favour of this interpretation, we found significant positive dependencies when only considering events for which an association involving an animate element was presented first but not when only considering events for which an association not involving an animate element was presented first for conditions in which there was a significant positive dependency.

Importantly, our findings cannot be attributed to differences in memory performance between conditions. Memory performance did, with few exceptions, not vary across conditions. Unsurprisingly, memory performance was lower for to-be-inferred associations in the open-loop conditions, resulting in an overall higher performance in the CL conditions in which all associations were shown in the learning phase. We did not find a difference in memory performance between events that include an animate element and events that do not. On the level of associations, there were generally also no differences between associations involving an animate element and associations not involving an animate element. Memory performance for associations not involving an animate element even tended to be higher in some conditions. We did thus not find an animacy effect in terms of memory performance. While the effect has been shown using a variety of test formats such as free recall (Bonin et al., 2015; Li et al., 2016; Madan, 2021; Nairne et al., 2013; Popp & Serra, 2016; VanArsdall et al., 2015), cued recall (DeYoung & Serra, 2021; Laurino & Kaczer, 2019), free recognition (Bonin et al., 2014; see also VanArsdall et al., 2013), and judgements of learning (DeYoung & Serra, 2021; Laurino & Kaczer, 2019), results using cued recall have been mixed (DeYoung & Serra, 2021; Kazanas et al., 2020; Laurino & Kaczer, 2019; Popp & Serra, 2016) and the effect has not yet been examined in the context of cued recognition tests which we used in the current research. In addition, Bonin et al. (2015) found that an imagery instruction improves performance for inanimate words but not for animate words. As we instructed participants to imagine the presented words as elements of a scene and to imagine them interacting in a meaningful manner, this instruction may have prevented the emergence of an animacy effect regarding memory performance by boosting memory performance for the inanimate elements. Considering the diluting effect of mental imagery on animacy effects, the potency of animacy in influencing the binding of event elements may actually be underestimated in Experiments 2 and 3.

Taken together, our findings suggest that binding structures may change depending on event characteristics and perceived task demands. While they do not clearly distinguish between an integrated and a hierarchical binding structure, they suggest animacy to influence the binding of event elements and hint at an influence of event structure awareness.

### Limitations

There are at least three potential limitations concerning the current research. First, due to the COVID-19 pandemic and the resulting limitations regarding lab-based data collection, all experiments were conducted online and took about 45 to 65 min to complete. Web-based studies naturally do not have the degree of experimental control that can be achieved in lab-based studies. However, several studies have shown comparable data quality for web- and lab-based studies (Armitage & Eerola, 2020; Bartneck et al., 2015; Dandurand et al., 2008; de Leeuw & Motz, 2016; Hilbig, 2016). A decrease in attention is also not necessarily found in web-based studies (Clifford & Jerit, 2014; Hauser & Schwarz, 2016) and the precision of stimulus timing of lab.js (Henninger et al., 2020), which was used for the implementation of our experiments, was found to be good (Anwyl-Irvine et al., 2021; Bridges et al., 2020). In addition, James et al. (2020) used the separated encoding paradigm in a web-based format before and found highly replicable effects. We too found the effect of a positive dependency when the encoding episode is coherent and events include an animate element in two out of three experiments, which is in favour of the robustness of the effect in web-based settings and sufficient data quality in our experiments.

Second, the separated encoding paradigm (Horner & Burgess, 2014; Horner et al., 2015) deviates to some extent from how events are “naturally” experienced, because temporal dependencies between event segments are reduced due to the interleaved presentation of learning trials referring to different events. However, the paradigm allows to manipulate the associative structure of event presentations, which is necessary when trying to distinguish between different binding structures, which was one of the goals of the current research. In addition, it allows to explore, for example, presentation order effects, such as whether dependency is higher for events for which an association involving an animate element was presented first than for events for which an association not involving an animate element was presented first.

Third, while we believe the newly proposed approach for modelling dependencies of the retrieval of event elements to be a substantial improvement over existing approaches, it has some limitations. First, it is somewhat limited in terms of the type of comparisons that can be conducted. Because the sampling distribution of the dependency index is unknown, it requires bootstrapping to draw statistical inferences. Thus, when comparing dependency indices of different conditions, only pairwise comparisons are currently possible. Second, floor or ceiling effects of memory performance may lead to an unreliable estimation of dependency indices, a problem that is also inherent to other measures. The results of the memory performance analysis, however, indicate that this was not an issue in our experiments. Third, if there are items that have no variance, the estimation of item parameters for these items is not possible. The risk of this to occur increases with smaller samples and more missing values. However, this was also not an issue in the current research. Fourth, while the modelling approach is rather robust against model misspecifications, model misspecifications may nevertheless lead to small shifts in dependency estimates and obtained *p* values. The same may be true for different sorting of items due to variability in item parameter estimation. When using parametric bootstrapping to obtain *p* values, these are to some degree also affected by Monte Carlo error. The Monte Carlo error can be reduced by increasing the number of bootstrap samples. We recommend to use at least 1,000 bootstrap samples (cf. Davison & Hinkley, 1997).

### Directions for future research

In terms of future research, it is necessary to conduct additional studies to obtain evidence distinguishing between an integrated and a hierarchical binding structure. We think that the separated encoding paradigm (Horner & Burgess, 2014; Horner et al., 2015) with systematic variations of the excluded associations as done in the current research is a useful paradigm to this end. It may not necessarily be the case that binding always occurs in the same way. On the contrary, our results suggest that binding may be influenced by several moderators. We deem it very important to identify and clarify such moderators in future research, a topic that is yet underrepresented in the literature. Identifying these moderators will help to exert more experimental control and to rule out additional explanations for observed or unobserved effects. As our results hinted at a role of awareness of event structures in the binding of event elements, future research could examine effects of varying event structures or task demands systematically. In addition, the role of animacy in the binding of event elements should be examined more closely. For example, if animacy exerts its role by making available an agent in the event, agency instead of animacy may be causal for the effects. Consequently, similar effects should be found when manipulating the agency of specific event elements. It may also prove fruitful to manipulate presentation order (i.e., whether an association involving an animate element is presented first or an association not involving an animate element is presented first) systematically, because our post hoc analysis on this matter suggested an effect of presentation order. Furthermore, because the results regarding specific recollection judgements were quite inconsistent, future research could try different adaptations of the remember–know paradigm to evaluate its suitability for more complex representations such as those that are the focus of the current research. Finally, the newly proposed approach for modelling dependencies of the retrieval of event elements warrants further systematic examination to identify other potential strengths and weaknesses and areas for improvement.

## Conclusion

In three experiments, we investigated whether the binding of event elements in episodic memory occurs in an integrated manner, in which event elements are bound into a unitary representation, or in a hierarchical manner, in which event elements are preferentially bound to particular elements. The experiments yielded inconsistent results which cannot clearly distinguish between an integrated and a hierarchical binding structure, which necessitates further research. However, the experiments yielded evidence that animacy influences the binding of event elements, a moderator that has not been previously considered. In addition, we identified event structure awareness, which may be affected by variability in event structure, as a potential additional moderator. Thus, the binding of event elements may vary based on several moderators such as animacy and perceived task demands. Finally, we provide a new approach for modelling dependencies of the retrieval of event elements in episodic memory which mitigates some limitations of previous approaches.

## Supplemental Material

sj-pdf-1-qjp-10.1177_17470218221096148 – Supplemental material for The binding structure of event elements in episodic memory and the role of animacy Click here for additional data file. Supplemental material, sj-pdf-1-qjp-10.1177_17470218221096148 for The binding structure of event elements in episodic memory and the role of animacy by Marcel R Schreiner, Thorsten Meiser and Arndt Bröder in Quarterly Journal of Experimental Psychology

## Notes

1 In the context of the current research, an item refers to a test trial in the memory test, which is a binary outcome (0 = distractor chosen, 1 = target chosen).

2 A finding that there is no stochastic dependency of the retrieval of event elements in non-coherent encoding episodes (open-loop structures) would be consistent with the interplay of two assumptions: integrated binding structures and the necessity of coherent encoding episodes. However, the latter assumption is not a necessary premise for integrated binding structures. Thus, a result pattern in which there is a dependency of the retrieval of event elements in non-coherent encoding episodes but this dependency does not vary as a function of the excluded association would still be consistent with an integrated binding structure, although it would violate the assumption of the necessity of coherent encoding episodes.

3 The factor association refers to the element pair being tested in the cued recognition test. The possible associations were animal–object, animal–location, and object–location. For example, a test trial in which the cue is an animal and the target is an object and a test trial in which the cue is an object and the target is an animal both test the association animal–object.

4 The Bayesian information criterion (BIC) for a given model M can be computed as BIC(M)=klog(n)−2log(L) , where *k* is the number of free parameters of the model, *n* is the number of observations, and *L* is the maximum likelihood of the model. Given two models $M_{0}$ and $M_{1}$ , one can approximate the Bayes factor in favour of $M_{1}$ as ${\mathrm{BF}}_{10}=\exp((BIC_{0}-BIC_{1})/2)$ . This BIC approximation can be considered to assume the unit information prior, which contains as much information as, on average, a single observation (see Raftery, 1995; Wagenmakers, 2007).

5 In the preregistration, we noted that we would draw item parameters from a standard normal distribution. However, estimating the parameters from the data allows to better account for differences in memory performance between conditions.

6 While we initially planned to compute one-tailed *p* values, we switched to two-tailed *p* values due to the (unexpected) occurrence of negative dependencies in the data, which warranted testing.

7 In the preregistration, we mentioned that we would calculate *p* values based on the mean of the difference values. However, this approach may lead to a too liberal criterion because it uses more item information than is available in the empirical data. Thus, we used the distribution of the individual difference values for computing *p* values instead.

8 We excluded participants with time lags larger than 1 min between screens in the learning or test phase except if this occurred only once and the lag was less than 5 min. For lags between the learning and test phase, we applied a less restrictive criterion because this part of the experiment also contained instructions. Regarding lags between the learning and test phase, we excluded participants with time lags larger than 3 min except if this occurred only once and the lag was less than 7 min. Larger time lags between screens are indicative of participants interrupting the experiment.

9 One participant did not give any remember responses and four participants did not give any know responses. These participants were excluded from the respective analyses, resulting in *n* = 148 for the analysis for remember responses and *n* = 145 for the analysis for know responses.

10 A value of 0 indicates no difference in odds between groups. Positive values indicate higher odds and negative values indicate lower odds in the first group than in the second group.

11 The number of events per condition was considered in the power analysis. Thus, given the suggested sample size, the modelling approach is sufficiently robust to the lower number of events per condition.

12 The loop conditions and associations were coerced into a common factor level because they differed between the animacy and non-animacy condition, and thus needed to be “equated” to jointly include them in the generalised linear mixed model.

13 Note that condition OL-ol (open loop with association object–location excluded) in Experiment 1 also yielded a significant positive dependency but was not included in the post hoc analysis of presentation order regarding animacy.
