# Supplementary material for: The binding structure of event elements in episodic memory and the role of animacy
Source: Q J Exp Psychol (Hove). 2022 Jun 9;76(4):705–30. doi: 10.1177/17470218221096148 (PMC10031638; doi:10.1177/17470218221096148)
Supplement: sj-pdf-1-qjp-10.1177_17470218221096148 – Supplemental material for The binding structure of event elements in episodic memory and the role of animacy [file sj-pdf-1-qjp-10.1177_17470218221096148.pdf]

Appendix A

Bifactor IRT Model

The bifactor IRT model we used extends the model in (1) by including additional latent traits λ for each event:

$$P(u_{ij} = 1) = \gamma_j + (1 - \gamma_j)\frac{e^{\alpha_j(\theta_i - \beta_j) - \alpha_{t(j)}\lambda_{it(j)}}}{1 + e^{\alpha_j(\theta_i - \beta_j) - \alpha_{t(j)}\lambda_{it(j)}}} \tag{A1}$$

where λ is the event-specific trait of person i for event *t(j)* to which item j belongs. Thus, each item loads on the general latent person trait and on an additional event-specific latent trait. Applying the same restrictions as in (2) reduces the model to:

$$P(u_{ij} = 1) = \frac{1}{6} + \frac{5}{6}\frac{e^{\theta_i - \beta_j - \lambda_{it(j)}}}{1 + e^{\theta_i - \beta_j - \lambda_{it(j)}}} \tag{A2}$$

The event-specific latent traits are mutually independent and independent to the general latent person trait θ. They exert their influence via their variances, with higher variances indicating a larger event-specific effect.

Appendix B

Dependency Analysis Using the Approach by Horner and Burgess

We also analysed the dependency of the retrieval of event elements using the approach outlined in Horner and Burgess (2013), using code provided by James et al. (2020). Because Horner and Burgess (2013) tested associations in both directions, resulting in six test trials per event, the approach had to be adapted to fit the current procedure, which tested associations in only one direction, resulting in three test trials per event. While Horner and Burgess (2013) identified test pairs based on a common cue or target element, we identified test pairs based on the occurrence of a common element in the tested associations. For example, for the animacy conditions this resulted in three test pairs: the trials testing animal – object and animal – location, the trials testing animal – object and object – location, and the trials testing animal – location and object – location. The approach yields participant-specific dependency estimates for each condition. We tested for the presence of dependency in each condition using one-sample t-tests against 0. We compared dependency across conditions using linear mixed models and performing planned comparisons (one-tailed testing). For the linear mixed models we report Bayes factors in favour of an effect obtained using BIC approximation (Raftery, 1995; Wagenmakers, 2007) and the change in marginal R^2 (Nakagawa et al., 2017). In Experiments 2 and 3, loop conditions were coerced for the linear mixed model as done for the memory performance analysis.

In Experiment 1 there were significant positive dependencies in all conditions (M_{CL} = 0.02, $t(148) = 3.41$, $p = .001$, $d = 0.28$; $M_{\text{OL-ao}} = 0.01$, $t(148) = 3.01$, $p = .003$, $d = 0.25$; $M_{\text{OL-al}} = 0.01$, $t(148) = 3.11$, $p = .002$, $d = 0.25$; $M_{\text{OL-ol}} = 0.03$, $t(148) = 5.27$, $p < .001$, d = 0.43). There was strong evidence against an effect of condition according to the linear mixed model analysis ($\text{BF}_{10} < 0.001$, $R^2_{\text{change}} = .01$). Planned comparisons revealed that the dependency in condition CL was not significantly higher compared to the open-loop conditions ($t(444)$ = -0.37, $p = .64$). However, the dependency in condition OL-ol was

significantly higher compared to conditions OL-ao and OL-al ($t(444) = 2.65$, $p = .004$).

These results are largely congruent with the ones from the main dependency analysis. They support Hypothesis 1, which stated that there is a stochastic dependency of the retrieval of event elements and Hypothesis 4, which stated that dependency varies as a function of the excluded association in non-coherent encoding episodes. The results are thus in favour of a hierarchical binding structure. Contrary to the main dependency analysis, the significant yet weak dependencies in conditions OL-ao and OL-al partially support Hypothesis 3, which stated that dependency is reduced but not eliminated in non-coherent encoding episodes.

In Experiment 2, there were significant positive dependencies in conditions OL-ao ($M = 0.01$, $t(212) = 2.29$, $p = .02$, $d = 0.16$) and OL-ol ($M = 0.01$, $t(212) = 2.68$, $p = .008$, $d = 0.18$) in the animacy condition, but no significant dependencies in conditions CL ($M = 0.00$, $t(212) = 0.56$, $p = .58$, $d = 0.04$) and OL-al ($M = 0.00$, $t(212) = 0.88$, $p = .38$, $d = 0.06$). There were no significant dependencies in the non-animacy condition ($M_{\text{CL}} = 0.01$, $t(212) = 1.44$, $p = .58$, $d = 0.10$; $M_{\text{OL-o}_{\text{tr}}\text{o}_{\text{to}}} = 0.00$, $t(212) = -0.02$, $p = .98$, $d = 0.00$; $M_{\text{OL-o}_{\text{tr}}\text{l}} = 0.00$, $t(212) = 1.00$, $p = .32$, $d = 0.07$; $M_{\text{OL-o}_{\text{to}}\text{l}} = 0.00$, $t(212) = 0.70$, $p = .48$, $d = 0.05$). There was strong evidence against main effects of animacy condition ($\text{BF}_{10} < 0.001$, $R^2_{\text{change}} = .001$) and loop condition ($\text{BF}_{10} < 0.001$, $R^2_{\text{change}} < .001$) and against an interaction ($\text{BF}_{10} < 0.001$, $R^2_{\text{change}} = .002$) according to the linear mixed model analysis. Planned comparisons revealed that the dependency in condition CL was not significantly higher compared to the open-loop conditions in the animacy condition ($t(1484) = -1.09$, $p = .86$) and in the non-animacy condition ($t(1484) = 0.86$, $p = .19$). The dependency in condition OL-ol was not significantly higher compared to conditions OL-ao and OL-al in the animacy condition ($t(1484) = 0.97$, $p = .17$). The dependency in condition OL-o_{to}l was not significantly higher compared to conditions OL-$\text{o}_{\text{tr}}\text{o}_{\text{to}}$ and OL-o_{tr}l in the non-animacy condition ($t(1484) = 0.23$, $p = .41$).

These results are largely congruent with the ones from the main dependency

analysis. While, contrary to the main dependency analysis, the dependency in condition OL-ao and OL-ol in the animacy condition reached significance, the effects were weak and the dependency in condition OL-ol was not significantly larger than in the other open-loop conditions. Like the results of the main dependency analysis, the result can neither clearly distinguish between an integrated and a hierarchical binding structure, nor do they provide evidence for a special role of animacy.

In Experiment 3, there was a significant positive dependency in condition CL in the animacy condition (M = 0.02, $t(130)$ = 2.99, p = .003, d = 0.26) but no significant dependencies in the open-loop conditions ($M_{\text{OL-ao}}$ = 0.01, $t(130)$ = 1.43, p = .15, d = 0.13; $M_{\text{OL-al}}$ = 0.00, $t(130)$ = 0.77, p = .44, d = 0.07; $M_{\text{OL-ol}}$ = 0.01, $t(130)$ = 1.48, p = .14, d = 0.13). There were no significant dependencies in the non-animacy condition (M_{CL} = 0.01, $t(121)$ = 0.88, p = .38, d = 0.08; $M_{\text{OL-o}_{\text{tr}}\text{o}_{\text{to}}}$ = 0.00, $t(121)$ = -0.85, p = .40, d = -0.08; $M_{\text{OL-o}_{\text{tr}}\text{l}}$ = -0.01, $t(121)$ = -1.35, p = .18, d = -0.12; $M_{\text{OL-o}_{\text{to}}\text{l}}$ = 0.00, $t(121)$ = 0.25, p = .80, d = 0.02). There was strong evidence against main effects of animacy condition (BF_{10} = 0.005, R^2_{change} = .007) and loop condition (BF_{10} < 0.001, R^2_{change} = .005) and against an interaction (BF_{10} < 0.001, R^2_{change} < .001) according to the linear mixed model analysis. Planned comparisons revealed that the dependency in condition CL was significantly higher compared to the open-loop conditions in the animacy condition ($t(753)$ = 1.81, p = .04) but not in the non-animacy condition ($t(753)$ = 1.38, p = .08). The dependency in condition OL-ol was not significantly higher compared to conditions OL-ao and OL-al in the animacy condition ($t(753)$ = 0.26, p = .40). The dependency in condition OL-o_{to}l was not significantly higher compared to conditions OL-$\text{o}_{\text{tr}}\text{o}_{\text{to}}$ and OL-o_{tr}l in the non-animacy condition ($t(753)$ = 1.02, p = .15).

These results are largely congruent with the main dependency analysis except for the non-significant dependencies in the open-loop conditions in the non-animacy condition, which were significantly negative in the main dependency analysis. The results support Hypothesis 1 but do not support Hypotheses 3 and 4. They are thus in favour of an

integrated binding structure. The results further support Hypothesis 5b, which stated that dependency does not vary as a function of the excluded association in non-coherent encoding episodes if events do not include an animate element, but do not support Hypothesis 5a, which stated that dependency varies as a function of the excluded association in non-coherent encoding episodes if events include an animate element. However, like the results of the main dependency analysis, the results suggest that animacy facilitates the binding of event elements if the encoding episode is coherent.

Appendix C

Power Analysis Description

Power analyses were conducted using Monte Carlo simulations. Data were drawn from the bifactor model in (A2) using 1000 replications. Differences between conditions were induced by specifying differences in the variances of event-specific latent traits. For example, a difference of 1 was considered a medium effect (cf. Glas et al., 2000; Wang et al., 2002). This value was subtracted from the event-specific trait variance of a reference condition. Latent factor variances of both the general person trait and the reference event-specific trait were based on the ones from a pilot study ($N = 27$; Experiment 1) or on the ones from Experiment 1 (Experiments 2 and 3). The pilot study was using the simultaneous encoding paradigm (Horner & Burgess, 2013, 2014) and yielded higher dependency estimates than the separated encoding paradigm (Horner et al., 2015; Horner & Burgess, 2014; see also James et al., 2020) used in the main experiments, thus leading to a higher baseline dependency in the power analysis. This makes the power analysis more conservative. Reference event-specific trait variances were 4 for the power analysis for Experiment 1 and 1 for the power analyses for Experiments 2 and 3. The power analyses required critical values to which the estimated differences in dependency could be compared to determine statistical significance. These critical values were obtained by conducting other Monte Carlo simulations. Data were again drawn from the bifactor model in (A2) using 1000 replications. However, in these simulations all event-specific traits had the same variance relative to the reference condition. Then, difference values were computed and the 5% and 95% quantiles were used as critical values in the power analyses (one-tailed testing). The procedure was thus similar to the parametric bootstrap. Power analyses were targeted at the predicted pattern of effects. Thus, for each replication it was determined whether all predicted differences were significant. Power was determined over a range of sample sizes with an increment of 10.

Appendix D

Memory Performance by Cue, Target, and Trial Type

Table D1

Mean (M) and Standard Deviation (SD) of Memory Performance by Cue, Target, and Trial Type in each Condition of Experiment 1

		Condition							
		CL		OL-ao		OL-al		OL-ol	
		M	*SD*	*M*	*SD*	*M*	*SD*	*M*	*SD*
Cue type	animal	0.53	0.50	0.42	0.49	0.45	0.50	0.52	0.50
	object	0.57	0.50	0.53	0.50	0.46	0.50	0.42	0.49
	location	0.53	0.50	0.44	0.50	0.54	0.50	0.43	0.49
Target type	animal	0.55	0.50	0.41	0.49	0.44	0.50	0.50	0.50
	object	0.56	0.50	0.53	0.50	0.46	0.50	0.43	0.50
	location	0.55	0.50	0.45	0.50	0.55	0.50	0.43	0.50
Trial type	inference	–	–	0.33	0.47	0.36	0.48	0.35	0.48
	no inference	0.55	0.50	0.53	0.50	0.55	0.50	0.51	0.50

Note. CL = closed loop, OL-ao = open loop with association animal – object excluded, OL-al = open loop with association animal – location excluded, OL-ol = open loop with association object – location excluded.

Table D2

Mean (M) and Standard Deviation (SD) of Memory Performance by Cue, Target, and Trial Type in each Condition of Experiment 2

Animacy condition			Loop condition							
			CL		OL-ao/$o_{tr}o_{to}$		OL-al/$o_{tr}l$		OL-ol/$o_{to}l$	
			M	*SD*	*M*	*SD*	*M*	*SD*	*M*	*SD*
Animacy	Cue type	animal	0.46	0.50	0.34	0.48	0.35	0.48	0.44	0.50
		object	0.45	0.50	0.45	0.50	0.34	0.48	0.34	0.47
		location	0.46	0.50	0.35	0.48	0.42	0.49	0.35	0.48
	Target type	animal	0.45	0.50	0.36	0.48	0.34	0.47	0.45	0.50
		object	0.48	0.50	0.44	0.50	0.33	0.47	0.35	0.48
		location	0.44	0.50	0.34	0.47	0.45	0.50	0.33	0.47
	Trial type	inference	–	–	0.25	0.43	0.24	0.43	0.24	0.43
		no inference	0.46	0.50	0.44	0.50	0.44	0.50	0.45	0.50
Non-animacy	Cue type	transport	0.42	0.49	0.35	0.48	0.32	0.47	0.42	0.49
		tool	0.44	0.50	0.45	0.50	0.33	0.47	0.34	0.48
		location	0.43	0.49	0.34	0.48	0.41	0.49	0.34	0.47
	Target type	transport	0.43	0.50	0.34	0.47	0.31	0.46	0.43	0.50
		tool	0.44	0.50	0.45	0.50	0.34	0.47	0.35	0.48
		location	0.42	0.49	0.35	0.48	0.41	0.49	0.33	0.47
	Trial type	inference	–	–	0.24	0.43	0.24	0.43	0.25	0.44
		no inference	0.43	0.50	0.45	0.50	0.41	0.49	0.42	0.49

Note. CL = closed loop, OL-ao = open loop with association animal – object excluded, OL-al = open loop with association animal – location excluded, OL-ol = open loop with association object – location excluded, OL-$o_{tr}o_{to}$ = open loop with association means of transportation – tool excluded, OL-$o_{tr}l$ = open loop with association means of transportation – location excluded, OL-$o_{to}l$ = open loop with association tool – location excluded, transport = means of transportation. When two loop conditions are separated by a slash (/) the first one refers to the animacy condition and the second one refers to the non-animacy condition.

Table D3

Mean (M) and Standard Deviation (SD) of Memory Performance by Cue, Target, and Trial Type in each Condition of Experiment 3

Animacy condition			Loop condition							
			CL		OL-ao/$o_{tr}o_{to}$		OL-al/$o_{tr}l$		OL-ol/$o_{to}l$	
			M	*SD*	*M*	*SD*	*M*	*SD*	*M*	*SD*
Animacy	Cue type	animal	0.49	0.50	0.39	0.49	0.38	0.49	0.48	0.50
		object	0.53	0.50	0.50	0.50	0.39	0.49	0.38	0.49
		location	0.52	0.50	0.40	0.49	0.49	0.50	0.39	0.49
	Target type	animal	0.50	0.50	0.39	0.49	0.37	0.48	0.48	0.50
		object	0.53	0.50	0.50	0.50	0.40	0.49	0.39	0.49
		location	0.51	0.50	0.40	0.49	0.49	0.50	0.38	0.48
	Trial type	inference	–	–	0.29	0.45	0.28	0.45	0.29	0.45
		no inference	0.51	0.50	0.50	0.50	0.49	0.50	0.48	0.50
Non-animacy	Cue type	transport	0.44	0.50	0.36	0.48	0.32	0.46	0.43	0.49
		tool	0.45	0.50	0.44	0.50	0.34	0.48	0.33	0.47
		location	0.46	0.50	0.34	0.47	0.42	0.49	0.31	0.46
	Target type	transport	0.44	0.50	0.34	0.47	0.30	0.46	0.44	0.50
		tool	0.48	0.50	0.49	0.50	0.33	0.47	0.34	0.47
		location	0.43	0.50	0.32	0.47	0.44	0.50	0.29	0.45
	Trial type	inference	–	–	0.22	0.41	0.22	0.41	0.20	0.40
		no inference	0.45	0.50	0.46	0.50	0.43	0.50	0.43	0.50

Note. CL = closed loop, OL-ao = open loop with association animal – object excluded, OL-al = open loop with association animal – location excluded, OL-ol = open loop with association object – location excluded, OL-$o_{tr}o_{to}$ = open loop with association means of transportation – tool excluded, OL-$o_{tr}l$ = open loop with association means of transportation – location excluded, OL-$o_{to}l$ = open loop with association tool – location excluded, transport = means of transportation. When two loop conditions are separated by a slash (/) the first one refers to the animacy condition and the second one refers to the non-animacy condition.
